# Supplementary material for: High-level production of nervonic acid in the oleaginous yeast Yarrowia lipolytica by systematic metabolic engineering
Source: Commun Biol. 2023 Nov 7;6:1125. doi: 10.1038/s42003-023-05502-w (PMC10630375; doi:10.1038/s42003-023-05502-w)
Supplement: Supplementary file 2 — Supplementary Information [file 42003_2023_5502_MOESM2_ESM.pdf]

High-level production of nervonic acid in the oleaginous yeast *Yarrowia lipolytica* by systematic metabolic engineering

Hang Su[#a,f], Penghui Shi[#a,d], Zhaoshuang Shen[#a,d], Huimin Meng[a†], Ziyue Meng[a,f], Xingfeng Han[a], Yanna Chen[b], Weiming Fan[b], Yun Fa[a,d,e], Chunyu Yang[c], Fuli Li*[a,d,e], and Shi'an Wang*[a,d,e]

a. *Key Laboratory of Biofuels, Qingdao Institute of Bioenergy and Bioprocess Technology, Chinese Academy of Sciences, Qingdao 266101, China.*
b. *Zhejiang Zhenyuan Biotech Co., LTD, Shaoxing 312365, China.*
c. *State Key Laboratory of Microbial Technology, Institute of Microbial Technology, Shandong University, Qingdao 266237, China.*
d. *Shandong Energy Institute, Qingdao 266101, China.*
e. *Qingdao New Energy Shandong Laboratory, Qingdao 266101, China.*
f. *University of Chinese Academy of Sciences, Beijing 100039, China.*

[#]These authors contributed equally to this work.

†*Present address, Qingdao Institute for Food and Drug Control, Qingdao 266073, China*

***Corresponding author:**

Shi'an Wang, Tel.: +86-532-80662682; Fax: +86-532-80662778.

E-mail: wangsa@qibebt.ac.cn

Fuli Li, Tel.: +86-532-80662656; Fax: +86-532-80662778.

E-mail: lifl@qibebt.ac.cn

Postal address: No.189 Songling Road, Laoshan District, Qingdao, Shandong Province, China, Postcode 266101

Supplementary Tables

Supplementary Table 1. The *Y. lipolytica* strains used in this study

Strain	Genotype	Source
po1g	*MATa, leu2-270, ura3-302::URA3, xpr2-3*	Yeastern
po1g-G3	po1g, *ACC1, DGA1, SCD, ΔKu70*	(Li et al., 2020)
YL-AtADS2	po1g, *AtADS2*	This work
YL-CgKCS	po1g, *CgKCS*	This work
YL-erCgKCS	po1g, *ER-CgKCS*	This work
YL-mtCgKCS	po1g, *MT-CgKCS*	This work
YL-peCgKCS	po1g, *PE-CgKCS*	This work
YL-erpeCgKCS	po1g, *ER-CgKCS*, *PE-CgKCS*	This work
YL-AtFAE1	po1g, *AtFAE1*	This work
YL-BtFAE1	po1g, *BtFAE1*	This work
YL-3FAEs	po1g, *AtFAE1*, *BtFAE1*, *CgKCS*	This work
YL-gfpKCS	po1g, *CgKCS-sfGFP*	This work
YL-gELOVE6	po1g, *gELOVE6*	This work
YL-MaOLE2	po1g, *MaOLE2*	This work
YL-3CgK	po1g, (*ER-CgKCS, MT-CgKCS, PE-CgKCS*)*2	This work
YL-3CgKE	YL-3CgK, *gELOVE6*	This work
YL-3CgKEM	YL-3CgKE, *gELOVE6*, *MaOLE2*	This work
YL-ΔFAD2	po1g, *FAD2::URA3*	This work
YLVL3	po1g-G3, *ER-CgKCS*, *MT-CgKCS*, *PE-CgKCS*	This work
YLVL6	YLVL3, *ER-CgKCS, MT-CgKCS, PE-CgKCS*	This work
YLVL7	YLVL6, rDNA::(*CgKCS-gElovl6-MaOLE2*)	This work
YLVL8	YLVL7, D17::(*CgKCS-gElovl6-MaOLE2*)	This work
YLVL10	YLVL8, *FAD2*::*CgKCS**2	This work
YLNA1	po1g-G3, rDNA::*CgKCS*	This work
YLNA3	YLNA1, *FAD2*::*CgKCS**2	This work
YLNA5	YLNA3, *TGL4*::*CgKCS**2	This work
YLNA6	YLNA5, *GSY1*::*CgKCS*	This work

YLNA7	YLNA6, *SNF1*::*CgKCS*	This work
YLNA8	YLNA7, *D17*::(*CgKCS-MaOLE2*)	This work
YLNA9	YLNA8, *PEX10*::(*YlINO2*)	This work
YLNA10	YLNA8, *PEX10*::(*MoGPAT-88*)	This work

Note: ER, endoplasmic reticulum; MT, mitochondria; PE, peroxisome.

Supplementary Table 2. Plasmids used in this study

Plasmid	Genotype	Source
pYLEX1	p*hp4d*, *LEU2*	Yeastern
pYLU	pYLEX1, *LEU2::URA3*	This work
pYL-AtADS2	pYLU, *TEFin-AtADS2*	This work
pYL-CgKCS	pYLU, *TEFin-CgKCS*	This work
pYL-erCgKCS	pYLU, *TEFin-ER-CgKCS*	This work
pYL-mtCgKCS	pYLU, *TEFin-MT-CgKCS*	This work
pYL-peCgKCS	pYLU, *TEFin-PE-CgKCS*	This work
pYL-AtFAE1	pYLEX1, *TEFin-AtFAE1*	This work
pYL-erAtFAE1	pYLEX1, *TEFin-ER-AtFAE1*	This work
pYL-peAtFAE1	pYLEX1, *TEFin-PE-AtFAE1*	This work
pYL-BtFAE1	pYLEX1, *TEFin-BtFAE1*	This work
pYL-erBtFAE1	pYLEX1, *TEFin-ER-BtFAE1*	This work
pYL-peBtFAE1	pYLEX1, *TEFin-PE-BtFAE1*	This work
pYL-gfpKCS	pYL-CgKCS, *TEFin-CgKCS-sfGFP*	This work
pYL-gELOVE6	pYLU, *YAT1-gELOVE6*	This work
pYL-MaOLE2	pYLU, *YAT1-MaOLE2*	This work
pYL-ΔFAD2	pYLU, *FAD2up-URA3-FAD2dn*	This work
pYL-2KCS	pYL-erCgKCS, *TEFin-MT-CgKCS*	This work
pYL-3sKCS	pYL-2KCS, *TEFin-PE-CgKCS*	This work
pYL-3grDNA	pYL-CgKCS,*rDNAup-CgKCS-gElovl6-MaOLE2-rDNAdn*	This work
pYL-3gD17	pYL-3grDNA,*D17up-CgKCS-gElovl6-MaOLE2-D17dn*	This work
pYL-2gFAD2	pYL-ΔFAD2, *(TEFin-CgKCS)* *2	This work
pYL-rDNA-CgKCS	pYL-3grDNA, *rDNAup-CgKCS -rDNAdn*	This work
pYL-2gTGL4	pYL-2gFAD2, *TGL4up-CgKCS-CgKCS-TGL4dn*	This work
pYL-GSY1-CgKCS	pYL-rDNA-CgKCS, *GSY1up-CgKCS-GSY1dn*	This work
pYL-SNF1-CgKCS	pYL-rDNA-CgKCS, *SNF1up-CgKCS-SNF1dn*	This work

pYL-D17-gMaolE2	pYL-rDNA-CgKCS, *D17up-CgKCS-MaolE2-D17dn*	This work
pYL-PEX10-YlINO2	pYL-rDNA-CgKCS, *PEX10up-YlINO2-PEX10dn*	This work
pYL-PEX10-YlINO4	pYL-rDNA-CgKCS, *PEX10up-YlINO4-PEX10dn*	This work
pYL-PEX10-Mo-35	pYL-rDNA-CgKCS, *PEX10up-MoDGAT-35-PEX10dn*	This work
pYL-PEX10-Mo-49	pYL-rDNA-CgKCS, *PEX10up- MoDGAT-49-PEX10dn*	This work
pYL-PEX10-Mo-88	pYL-rDNA-CgKCS, *PEX10up-MoGPAT-88-PEX10dn*	This work
pYL-PEX10-Mo-90	pYL-rDNA-CgKCS, *PEX10up-MoGPAT-90-PEX10dn*	This work

Supplementary Table 3. Sequences of synthetic genes used in this study

**Optimized gene sequences toward *Y. lipolytica* and the original sources**
**> *AtADS2*, originated from *Arabidopsis thaliana***<br>ATGTCTGTGACCTCTACCGTGGAGGAGAACCACCAGAAGAACCCCTCTACCCCTGCTGCTGTGGAGGAAAAGA<br>AGAAGCGACGATGGGTGTTCTGGGATCGACGATGGCGACGACTGGACTACGTGAAGTTCTCTGCCTCTTTCACC<br>GTGCACTCTCTGGCTCTTCTTGCCCCCTTCTACTTCACTTGGTCTGCCCTGTGGGTGACCTTCCTGTTCTACACCA<br>TTGGCGGCCTGGGCATTACTGTGTCTTACCACCGAAACCTGGCCCACCGATCTTTCAAGGTGCCCAAGTGGCTG<br>GAATACCTGCTGGCTTATTGTGCCCTGCTGGCCATTCAAGGTGACCCTATCGACTGGGTGTCTACCCACCGATACC<br>ACCACCAGTTCACCGACTCTGAACGAGATCCCCACTCTCCTAAAGAGGGCTTCTGGTTCTCTCACCTGCTGTGG<br>ATCTACGACTCTGCCTACCTGGTGTCTAAATGCGGCCGACGAGCCAATGTGGAGGATCTGAAGCGACAGTGGTT<br>CTACCGATTCCTGCAGAAGACCGTGCTGTTCCACATTCTTGGCCTGGGCTTCTTCCTGTTTTACCTGGGCGGCATG<br>TCTTTTGTGACCTGGGGAATGGGAGTGGGAGCTGCCCTGGAAGTTCATGTGACCTGCCTGATCAACTCTCTGTGC<br>CACATCTGGGGAACCCGAACCTGGAAAACCAACGACACCTCTCGAAACGTGTGGTGGCTGTCTGTGTTCTCTTT<br>CGGCGAGTCTTGGCACAACAACCACCACGCCTTCGAGTCTTCTGCTCGACAGGGACTGGAATGGTGGCAGATCG<br>ACATCTCTTGGTACATCGTGCGATTCTTCGAGATCATCGGCCTGGCCACCGATGTTAAAGTGCCCACCGAAGCCC<br>AACGAAGACGAATGGCCATCGTGAGAAAGGACGAGCTGTGA
**> *AtFAE1*, originated from *Arabidopsis thaliana***<br>ATGACCTCTGTGAACGTGAAGCTGCTGTACCGATACGTGCTGACCAACTTCTTCAACCTGTGCCTGTTTCCCCTG<br>ACTGCCTTTCTTGCCGGAAAAGCCTCTCGACTGACCATCAACGACCTGCACAACTTCCTGTCTTACCTGCAGCAC<br>AACCTGATCACCGTGACCCTGCTGTTTGCCTTCACCGTGTTCGGACTGGTGCTGTACATTGTGACCCGACCCAAC<br>CCTGTGTATCTGGTGGACTACTCTTGTTACCTGCCCCCCCCTCACCTTAAAGTGTCTGTGTCTAAGGTGATGGACA<br>TCTTCTACCAGATCCGAAAGGCCGACACCTCTTCTAGAAACGTGGCCTGCGACGACCCTTCTTCTCTGGACTTCC<br>TGCGAAAGATCCAGGAGAGATCTGGCCTGGGCGACGAAACTTACTCTCCCGAAGGCCTGATTCATGTGCCTCCC<br>CGAAAAACTTTCGCCGCCTCTAGAGAAGAGACCGAGAAGGTGATTATCGGCGCCCTGGAGAACCTGTTCGAGA<br>ACACCAAGGTGAACCCCCGAGAGATCGGCATTCTGGTGGTGAACTCTTCTATGTTCAACCCCACCCCCTCTCTTT<br>CTGCCATGGTGGTGAACACCTTCAAGCTGCGATCTAACATCAAGTCTTTCAACCTGGGCGGCATGGGATGTTCTG<br>CTGGAGTGATCGCCATCGACCTGGCTAAGGACCTGCTGCACGTGCACAAGAACACCTACGCCCTGGTGGTGTCT<br>ACCGAGAACATCACCCAGGGCATCTACGCTGGCGAGAACCGATCTATGATGGTGTCTAACTGCCTGTTCAGAGTT<br>GGAGGAGCCGCCATTCTGCTGTCTAACAAGTCTGGCGACCGACGATCTAAGTACAAGCTGGTGCACACCGT<br>GCGAACTCATACTGGCGCTGACGACAAGTCTTTCCGATGCGTGCAGCAGGAGGATGATGAGTCTGGCAAGATCG<br>GCGTGTGCCTGTCTAAGGACATCACCAACGTGGCTGGCACTACCCTGACTAAGAACATCGCCACTCTGGGCCCT<br>CTGATTCTGCCTCTGTCTGAGAAGTTCCTGTTCTTCGCCACCTTCGTGGCCAAGAAGCTGCTGAAGGACAAGATC<br>AAGCACTACTACGTGCCCGACTTCAAACTTGCCGTGGACCACTTTTGCATCCATGCTGGCGGCAGAGCTGTTATT<br>GACGAGCTGGAGAAGAACCTTGGCCTGTCTCCCATTGATGTGGAGGCCTCTCGATCTACCCTTCACCGATTCGGC<br>AACACCTCTTCTTCTTCTATCTGGTACGAGCTGGCCTACATCGAAGCCAAGGGCCGAATGAAGAAGGGCAACAA<br>GGCCTGGCAAATTGCTCTGGGCTCTGGCTTCAAGTGCAACTCTGCCGTGTGGGTGGCTCTGAGAAACGTGAAGG<br>CCTCTGCCAATTCTCCTTGGCAGCACTGCATTGACCGATACCCCGTGAAGATCGACTCTGACCTGTCTAAGTCTA<br>AGACCCACGTGCAGAACGGCCGATCTTAA
**> *BtFAE1*, originated from *Brassica tournefortii***<br>ATGACCTCTGTGAACGTGAAGCTGATCTACCACTACGTGATCACCAACCTGTTCAACCTGTGCTTCTTCCCCCTG<br>ACTGCTATCGTTGCCGGCAAAGCTTCTCGACTGACCATTGACGACCTGCACCACCTGTACTACTCTTACCTGCAG<br>CACAACCTGATTACCATCGCCCCCCTGTTTGCCTTTACCGTGTTCGGCTCTGTGCTGTACATTGCCACCCGACCTA<br>AGCCTGTGTACCTGGTGGAGTACTCTTGTTACCTGCCCCCCACTCACTGTCGATCTTCTATCTCTAAGGTGATGGA

CATCTTCTACCAGGTGCGAAAGGCCGACCCCTCTAGAAATGGCACCTGCGACGACTCTTCTTGGCTGGACTTCCT
GCGAAAGATCCAGGAGCGATCTGGCCTGGGAGATGAGACTCATGGCCCTGAGGGACTGCTTCACGTGCCTCCCA
GAAAAACCTTTGCCGCCGCTAGAGAAGAAACCGAGAAGGTGATTATCGGCGCCCTGGAGAACCTGTTCGAGAA
CACCAAGGTGAACCCCAAGGACATCGGCATCCTGGTGGTGAACTCTTCTATGTTCAACCCCACCCCCTCTCTTTC
TGCCATGGTGGTGAACACCTTCAAGCTGCGATCTAACGTGCGATCTTTCAACCTGGGAGGCATGGGATGTTCTGC
TGGCGTGATCGCTATTGACCTGGCCAAAGACCTGCTGCACGTGCACAAGAACACCTACGCCCTGGTGGTGTCTA
CCGAGAACATCACCTACAACATCTACGCCGGCGACAACAAGTCTATGATGGTGTCTAACTGCCTGTTCCGAGTGG
GAGGAGCCGCTATTCTGCTGTCTAACAAGCCCCGAGACCGACGACGATCTAAGTACGAGCTGGTGCACACCGTG
CGAACTCATACCGGAGCTGACGACAAGTCTTTCCGATGCGTGCAACAGGGCGATGACGAGTCTGGAAAGACCG
GCGTGTCTCTGTCTAAGGACATCACCGACGTGGCTGGAAGAACCGTGAAGAAGAACATCTCTACCCTGGGCCCT
CTGATTCTGCCTCTGTCTGAGAAGCTGCTGTTCTTCGTGACCTTCATGGGCAAGAAGCTGTTCAAGGACAAGATC
AAGCACTACTACGTGCCCGACTTCAAGCTTGCCATCGACCACTTCTGTATCCACGCTGGCGGACGAGCTGTGATT
GATGTGCTGGAGAAGAACCTTGGACTGGCCCCTATTGATGTGGAGGCCTCTCGATCTACCCTTCACCGATTCGGC
AACACCTCTTCTTCTTCTATCTGGTACGAGCTGGCCTACATCGAAGCTAAGGGCCGAATGAAGAAGGGCAACAA
GGTGTGGCAAATCGCTCTGGGCTCTGGCTTCAAGTGCAACTCTGCCGTTTGGGTGGCCCTGAGAAATGTGAAGG
CCTCTCGAAAGTCTCCTTGGGAGCACTGCATTGACCGATACCCCGTGAAGATCGACTACGACTCTGCCAAGTCTG
AGGTGAGAGTGCAGAACGGCCGATCTTAA

> *CgKCS*, originated from *Cardamine graeca*

ATGACCTCTATCAACGTGAAGCTGCTGTACCACTACGTGCTGACCAACTTCTTCAACCTGTGCCTGTTCCCCCTG
ACTGCTTTTCCTGCCGGCAAGGCTTCTCAACTGACCACCAACGACCTGCACCACCTGTACTCTTACCTGCACCAC
AACCTGATCACCGTGACCCTGCTGTTTGCCTTCACCGTGTTCGGCTCTATCCTGTACATCGTGACCAGACCCAAG
CCTGTGTACCTGGTGGACTACTCTTGTTACCTGCCCCCCAGACATCTGTCTTGCGGCATCTCTCGAGTGATGGAG
ATCTTCTACGAGATCCGAAAGTCTGACCCCTCTCGAGAGGTGCCTTTCGACGACCCTTCTTCTCTGGAGTTCCTG
CGAAAGATCCAGGAGCGATCTGGACTGGGAGACGAGACTTATGGACCCCAGGGACTGGTTCATGATATGCCCCT
GCGAATGAATTTTGCCGCCGCCCGAGAAGAAACTGAGCAGGTGATCAACGGAGCCCTGGAGAAGCTGTTCGAG
AACACCAAGGTGAACCCCCGAGAGATTGGCATCCTGGTGGTGAACTCTTCTATGTTCAACCCCACCCCCTCTCTT
TCTGCCATGGTGGTGAACACCTTCAAGCTGCGATCTAACATCAAGTCTTTCTCTCTGGGCGGCATGGGATGTTCT
GCCGGCATCATCGCTATCGACCTGGCTAAGGACCTGCTGCACGTGCACAAGAACACCTACGCCCTGGTGGTGTC
TACCGAGAACATCACCCACTCTACCTACACCGGCGACAACCGATCTATGATGGTGTCTAACTGCCTGTTCAGAAT
GGGCGGAGCCGCCATTCTGCTGTCTAACAAGGCTGGCGACCGACGACGATCTAAGTACAAGCTGGCCCACACTG
TGAGAACTCACACCGGCGCTGACGATCAGTCTTTCCGATGCGTGCGACAGGAGGATGATGACAGAGGCAAGATC
GGCGTGTGCCTGTCTAAAGACATCACCGCCGTGGCCGGAAAAACCGTGACCAAGAACATCGCTACCCTGGGAC
CTCTTGTGCTGCCTCTGTCTGAGAAGTTCCTGTACGTGGTGTCTCTGATGGCCAAGAAGCTGTTCAAGAACAAG
ATCAAGCACACCTACGTGCCCGATTTCAAGCTGGCCATCGACCACTTCTGCATCCATGCTGGCGGAAGAGCTGTG
ATTGACGTGCTGGAGAAGAACCTGGCCCTGTCTCCTGTGGATGTGGAGGCTTCTCGATCTACCCTGCACCGATTC
GGCAACACCTCTTCTTCTTCTATCTGGTACGAGCTGGCCTACATCGAAGCCAAGGGCCGAATGAAGAAGGGCAA
CAAGGTGTGGCAAATCGCCATCGGCTCTGGCTTCAAGTGCAACTCTGCCGTTTGGGTGGCCCTGTGTAATGTGA
AGCCCTCTGTGAACTCTCCTTGGGAGCACTGCATTGACAGATACCCCGTGGAGATCAACTACGGCTCTTCTAAGT
CTGAGACCCGAGCCCAAAACGGCCGATCTTAA

> sfGFP

GGCGGAGGCGGCGGAGGCGGAGGCGGAGGCAGAAAGGGTGAGGAGCTGTTTACAGGCGTTGTGCCCATCCTT
GTGGAGCTTGACGGCGATGTCAACGGTCATAAGTTCTCCGTTCGAGGAGAAGGCGAGGGTGATGCCACCAACG
GAAAGCTCACCCTTAAGTTCATCTGCACTACCGGTAAGCTGCCCGTGCCTTGGCCTACCCTGGTCACCACTCTCA
CCTATGGTGTGCAGTGCTTCGCCCGATACCCCGATCACATGAAGCAGCATGACTTTTTTAAGTCTGCCATGCCTGA

AGGCTACGTCCAGGAGCGAACCATCTCTTTCAAGGATGATGGAACCTACAAGACTCGAGCTGAGGTCAAGTTTG<br>AGGGTGATACTCTGGTCAACCGAATCGAGCTGAAGGGAATTGATTTTAAGGAGGATGGAAACATCCTCGGTCAC<br>AAGCTCGAGTACAACTTCAACTCCCACAACGTCTACATCACCGCTGATAAGCAGAAGAACGGAATTAAGGCTAA<br>CTTCAAGATTCGACACAATGTCGAAGACGGTTCCGTTCAGCTCGCTGACCACTACCAGCAGAACACTCCTATTG<br>GTGACGGACCCGTCCTGCTGCCCGACAACCATTACCTCTCGACCCAGTCGGTCCTTTCTAAGGACCCTAACGAG<br>AAGCGAGACCACATGGTCCTTCTGGAGTTCGTCACTGCCGCTGGAATTACCCACGGCATGGATGAGCTTTACAA<br>GTAA
**> *rELO2*, originated from *Rattus norvegicus***<br>ATGAACATGTCTGTGCTGACCCTGCAGGAGTACGAGTTCGAGAAGCAGTTCAACGAGAACGAGGCCATTCAGTG<br>GATGCAGGAGAACTGGAAGAAGTCCTTCCTGTTCTCCGCCCTGTACGCCGCCTTCATTTTCGGCGGCCGACACCT<br>GATGAACAAGCGAGCCAAGTTCGAGCTGCGAAAGCCCCTGGTCCTGTGGTCTCTGACCCTGGCCGTCTTCTCTA<br>TCTTCGGTGCCCTGCGAACCGGTGCCTACATGCTGTACATTCTGATGACCAAGGGCCTGAAGCAGTCCGTCTGTG<br>ACCAGTCCTTCTACAACGGTCCCGTCTCTAAGTTCTGGGCCTACGCCTTCGTCCTGTCCAAGGCCCCCGAGCTGG<br>GTGACACCATTTTCATCATTCTGCGAAAGCAGAAGCTGATTTTCCTGCACTGGTACCACCACATTACCGTCCTGCT<br>GTACTCCTGGTACTCCTACAAGGACATGGTCGCCGGCGGTGGCTGGTTCATGACCATGAACTACGGCGTGCACG<br>CCGTCATGTACTCCTACTACGCCCTGCGAGCCGCCGGCTTCCGAGTCTCTCGAAAGTTCGCCATGTTCATTACCCT<br>GTCTCAGATTACCCAGATGCTGATGGGTTGTGTCATCAACTACCTGGTCTTCAACTGGATGCAGCACGACAACGA<br>CCAGTGTTACTCCCACTTCCAGAACATTTTCTGGTCCTCCCTGATGTACCTGTCTTACCTGCTGCTGTTCTGCCAC<br>TTCTTCTTCGAGGCCTACATTGGTAAAGTGAAGAAGGCCACCAAGGCCGAGTAA
**> *CpLCE*, originated from *Cryptosporidium parvum***<br>ATGATCATCAAGAACAACAACAACGGCTACTTCATTGACAACGTCAACAAGATCTGGGAGATCCCCATCAACTCT<br>GAGCACATGGACATCCTGAAGGAGATCCCCTGGTTCAAGTACCTGACCCTGCCCATTGAGCGAAACTGGAACGG<br>CATGAAGCTGTTCCTGTGGACCAACGACAACTACTACCTGGCCCACACCATCTGCATCATCTACGCCTTCTTCATC<br>TACTTCGGTCCCAAGATCATGGAGAAGCGAAAGCCCTTCAAGCTGGAGAAGCCCCTGAAGTACTGGAACCTGTT<br>CCTGGCCCTGTTCTCTTTCATCGGCACCCTGCGACTGATGCCCTACGTGCTGACCAACCTGATTAAGTACGGCTTC<br>GTCTCTTCTATCTGCTCCCCCCCCATTGCCCCCCTGACCAAGGGCCCTGCCGGCCTGTGGCTGTCTCTGTTCATCT<br>ATTCCAAGTACATTGAGCTGATCGACACCTTCTTCATCATTGCCCGAAAGAAGTCTCTGTCCTTCCTGCACTGGTT<br>CCACCACCTGACCGTGCTGCTGTACACCTGGGACGCCTACGTCTGTTGTCAGACCATTGGTGTCTTCTTCTGTGC<br>CATTAACTACTTCGTGCACTCCATCATGTACTTCTACTACTACCTGTCCTCCTGTGGCAAGCGACCCAAGTGGGGT<br>ATGATCATTACCATTCTGCAGATCGTCCAGATGATTATTGGCACCATTCTGACCACCTCCGGTATGTACTACTCTTA<br>CAAGCACCCCTTCGCCAACGTGTTCCCCGTCGAGTACCTGTCCCAGCCCCTGAAGGTCGGTTGCCACTTCATCCG<br>AACCAACGGTGTCTTCGCCTGCCTGATGTACATCTCTTACTTCGCCCTGTTCTTCGACTTCTTCATCAAGCGATAC<br>ATCACCAAGGGCACCCCCCTGGCCGAGTGGGTCACCGCTAACAAGAAGCCCACCAAGCGAGACTAA
**> *gELOVL6*, originated from *Capra hircus***<br>ATGTCTGTGCTGACCCTGCAGGAGTACGAGTTCGAGAAGCAGTTCAACGAGAACGAGGCCATCCAGTGGATGC<br>AGGAGAACTGGAAGAAGTCTTTCCTGTTCTCTGCCCTGTACGCCGCCTTCGTCTTCGGCGGCCGACACCTGATG<br>AACAAGCGAGCCAAGTTCGAGCTGCGAAAGCCCCTGGTGCTGTGGTCTCTGACCCTGGCCGTGTTCTCCATTTT<br>CGGCGCCCTGCGAACCGGCGCCTACATGGTCTACACCGTGATGACCAAGGGCCTGAAGCACTCCGTCTGTGACC<br>AGGGCTTCTACAACGGCCCCGTCTCCAAGTTCTGGGCCTACGCCTTCGTCCTGTCTAAGGCCCCCGAGCTGGGT<br>GACACCATCTTCATTATCCTGCGAAAGCAGAAGCTGATTTTCCTGCACTGGTACCACCACATTACCGTCCTGCTGT<br>ACTCCTGGTACTCCTACAAGGACATGGTGGCCGGCGGTGGTTGGTTCATGACCATGAACTACTCTGTGCACTCTG<br>TGATGTACTCCTACTACGCCCTGCGAGCCGCCGGCTTCCGAGTCTCTCGAAAGTTCGCCATGTTCATCACCCTGT<br>CCCAGATTCTGCAGATGCTGGTGGGCTGTGTCATTAACTACCTGGTGTTCCAGTGGATGCAGCACGACCAGTGCC

ACTCCCACTTCCAGAACATCTTCTGGTCCTCTCTGATGTACCTGTCCTACTTCGTCCTGTTCTGCCACTTCTTCTTC
GAGGCCTACATCGGCTCTAAGATGCGAAAGGCCACCAAGGCCGACTAA

> D9DMB, originated from *Cunninghamella echinulata*

ATGTCCTCCGTGGTCGAGACCTGTAAGACCCTGCCCGTGCAGGCCTCTCGACTGGTGCCCACCGCCGTCAAGGA
GGAGGTCCCCACCGACCACAACGTCCCCGACAACTACGTGTCCTGGACCCTGAAGAACCAGAAGCCCCTGCCC
CCCATCGTGCTGGCCAACCTGCTGCAGAACATCGAGTGGCTGACCTTCATCATTCTGACCATCACCCCCTCCATC
GCCATCTACGGTCTGTTCACCGTCAACATGCAGTGGAAGACCTTCGTGTGGTCTGTCATGTACTACTTCATCACC
GGCCTGGGCATCACCGCCGGCTACCACCGACTGTGGTCTCACCGATCCTACAACGCCTCTAAGCCCCTGCAGTAC
TTCCTGGCCGTCGCCGGCACCGGTGCCGTTGAGGGTTCCATCAAGTGGTGGTGCCGAAAGCACCGAGCCCACC
ACCGATACACCGACACCGACCTGGACCCCTACAACGCCAACAAGGGTTTCTTCTTCTCCCACGTGGGCTGGATG
CTGGTCAAGCCCCGAGACAAGCCCGGTGTGGCCGACATTTCTGACCTGCGAAAGTCCGAGGTCATTCAGTGGCA
GCACCGATGGTACGTGTGGCTGATTGTGGGTGCCGGTCTGCTGCTGCCCACCGTGGTCGCTGGTCTGGGCTGGG
GAGACTGGGCCGGTGGTTTCTTCTTTGCCGGTGCCGCCCGACTGACCTTCGTGCACCACTCTACCTTCTGTGTCA
ACTCTCTGGCCCACTGGCTGGGTGAGACCCCCTTCGACGACAAGCACTCTCCCCGAGACCACTTCATTACCGCC
CTGGTCACCGTGGGCGAGGGCTACCACAACTTCCACCACCAGTTCCCCATGGACTACCGAAACGCCATTCGATG
GTACCAGTACGACCCCACCAAGTGGTTCATTTGGATGTGCTCCAAGTTCGGCCTGGCCTCTCACCTGAAGGTCTT
CCCCGAGAACGAGGTGCGAAAGGGCGAGCTGACCATGCAGCTGAAGAAGCTGCGAGAGACCCAGGACGTGCT
GACCTGGGCCCCCGACGTGGAGGGACTGCCTGTGATTTCTTGGGAGTCCTTCTCTGAGCAGTCCGTCACCCGAC
CCCTGATCATCGTCGCCGGCTTCATCCACGACGTCTCTGACTTCATCGACGAGCACCCCGGTGGCCGACACCTGA
TTATTAAGTACATTGGTAAAGACGCCACCCCCGCCTTCTTCGGCGGCGTTTACGACCACTCCAACGCCGCCCACA
ACCTGCTGTCCATGAAGCGAGTCGGTGCCCTGCACGGCGGCATCCAGCACGGACTGCAGGACATGTCTATTCCC
CCCGCCCAGCGACTGCGAATCGCCCGATACAACGAGCTGGCCGGCTCTCCCTACAACTCCTCTACCGCCACCTCT
GACTCTGAGGGTATTCTGGGTTAA

> CeFAT6, originated from, *Caenorhabditis elegans*

ATGACCGTCAAGACCCGATCCAACATTGCCAAGAAGATCGAGAAGGACGGTGGCCCCGAGACCCAGTACCTGG
CCGTGGACCCCAACGAGATCATTCAGCTGCAGGAGGAGTCTAAGAAGATCCCCTACAAGATGGAGATCGTCTGG
CGAAACGTGGCCCTGTTCGCCGCCCTGCACTTCGCCGCTGCCATCGGACTGTACCAGCTGATTTTCGAGGCCAA
GTGGCAGACCGTGATTTTCACCTTCCTGCTGTACGTCTTCGGTGGTTTCGGCATTACCGCCGGTGCCCACCGACT
GTGGTCTCACAAGTCCTACAAGGCCACCACCCCCATGCGAATTTTCCTGATGATCCTGAACAACATTGCCCTGCA
GAACGACGTCATTGAGTGGGCCCGAGACCACCGATGTCACCACAAGTGGACCGACACCGACGCCGACCCCCAC
AACACCACCCGAGGTTTCTTCTTCGCCCACATGGGCTGGCTGCTGGTGCGAAAGCACCCCCAGGTGAAGGAGC
AGGGTGCCAAGCTGGACATGTCCGACCTGCTGTCTGACCCCGTCCTGGTGTTCCAGCGAAAGCACTACTTCCCC
CTGGTCATCCTGTGTTGTTTCATTCTGCCCACCATTATCCCCGTCTACTTCTGGAAGGAGACCGCCTTCATCGCCT
TCTACACCGCCGGTACCTTCCGATACTGCTTCACCCTGCACGCCACCTGGTGTATCAACTCCGCCGCCCACTACTT
CGGTTGGAAGCCCTACGACTCCTCCATTACCCCCGTGGAGAACGTGTTCACCACCATCGCCGCCGTCGGCGAGG
GCGGTCATAACTTCCACCACACCTTCCCCCAGGACTACCGAACCTCCGAGTACTCTCTGAAGTACAACTGGACC
CGAGTGCTGATCGACACCGCCGCCGCCCTGGGACTGGTGTACGACCGAAAGACCGCCTGTGACGAGATCATCGG
CCGACAGGTGTCTAACCACGGCTGTGACATCCAGCGAGGTAAATCTATTATGTAA

> *MaOLE2*, originated from *Mortierella alpine*

ATGGCCACCCCCCTGCCTCCTACCTTCACCGTGCCCGCCTCCTCCACCGAGACCCGACGAGACCCCCTGCCCCA
CGACGTGCTGCCTCCTCTGTTCAACGGTGAGAAGGTCAACATCCTGAACATTTGGAAGTACCTGGACTGGAAGC
ACGTCATCGGCCTGCTGGTGACCCCCCTGGTCGCCCTTTACGGTATGTGTACCACCGAGCTGCACACCAAGACCC
TGGTCTGGTCCATCGTGTACTACTTCGCCACCGGCCTGGGTATCACCGCCGGTTACCACCGACTGTGGGCCCACC
GAGCCTACAACGCCGGTCCCGCTATGTCCTTCGCCCTGGCCCTGTTCGGCGCCGGAGCTGTTGAGGGCTCTATCA

AGTGGTGGTCCCGAGGCCACCGAGCCCACCACCGATGGACCGACACCGAGAAGGACCCCTACTCCGCCCACCG
AGGTGTGTTCTACTCTCACCTGGGTTGGCTGCTGATCAAGCGACCCGGTTGGAAGATTGGTCACGCCGACGTCG
ACGACCTGAACAAGAACCCCCTGGTCCAGTGGCAGCACAAGCACTACCTGATCCTGGTCATTCTGATGGGTCTG
GTGTTCCCCACCGCCGTCGCCGGTCTGGGATGGGGTGACTGGCGAGGTGGCTACTTCTACGCCGCCATTCTGCG
ACTGATCTTCGTGCACCACGCCACCTTCTGCGTGAACTCTCTGGCCCACTGGCTGGGCGACGGCCCTTTCGACG
ACCGACACACCCCCCGAGACCACTTCATCACCGCCTTCCTGACCCTGGGCGAGGGTTACCACAACTTCCACCAC
CAGTTCCCCCAGGACTACCGATCCGCCATCCGATTCTACCAGTACGACCCCACCAAGTGGCTGATTGCCACCTGC
GCCTTCTTCGGTTTCGCCTCTCACCTGAAGACCTTCCCCGAGAACGAGATTAAGAAGGGCAAGCTGCAGATGAT
TGAGAAGGAGGTGCTGGAGAAGAAGACCAAGCTGCAGTGGGGTACCCCCATCGCCGACCTGCCCATCCTGTCC
TTCGAGGACTTCCAGCACGCCTGCAAGAACGACCGAAAGCAGTGGATCCTGCTGGAGGGCGTCGTCTACGACG
TCGCCGACTTCATGACCGAGCACCCCGGTGGCGAGAAGTACATCAAGATGGGCGTGGGTAAAGACATGACCTCC
GCCTTCAACGGCGGTATGTACGACCACTCTAACGCCGCCCGAAACCTGCTGTCTCTGATGCGAGTCGCCGTCGTC
GAGTTCGGTGGCGAGGTCGAGGCCCAGAAGTCCCGACCCTCTGTGACCGTGTACGGCGACCACTCTAAGGAGG
AGTAA

> ***MoDGAT2*(Maole_010035) , originated from *Malania oleifera***

ATGGACGGCTCTTCGCAAATCAAGGAGCTTTCATGTCTTGTGAAATTTGTAGAAGAAACTGTGAGGATAGAGCAT
GCTTCAAACCCAAATAAGCCAGTTTATTTGGTAGGGGATTCTTTTGGTGGATGCTTGGCACTTGCTGTTGCTGCTC
GTAATCCTACATTTGATCTGGTTCTGATTTTAGTCAATCCAGCAACGTCATTTGGCAGGTCACAGCTGCAACCTCT
GCTGCCTGTCTTAGAGGCTTTGCCGGATGGGCTTCATTTCACTGTTCCTTATCTCCTTAGCTTTATCATGGGTACGT
GTTCTGCTGCTTTGAAGCCTTACCGTGATCCACTGAAGTTGGCAATGGTCAATATTGAAACTAGGTTTCCTCCTGC
ACTAGTACTAGAGCAATTGTCTGGCAACCTCACAGCTTTGCTACCACGCCTTTCTGGCTTGGCTAATATTATACCG
AAGGAGACTCTTCTTTGGAAGCTAAAGCTCCTAAAATCAGCTGCTGCTTATGCTAATTCACGTCTCCATGCTGTTA
AAGCTGAAGTACTTGTTCTGGCTAGTGGCAGTGATAACATGCTACCTAGCAAAGATGAAGCTGAACGCCTTTCAC
GTTCATTACAGAATTGCATAGTTCGTCTCTTTAAGGACAATGGGCATACCCTTCTATTGGAAGATGGCTATAGTCTG
CTGACCATCATTAAATGTACTTCCAAATACCGTTGTTCAAGGAAGCATGATTTCATCACGGATTTTCTGCCTCCAA
GTATGTCTGAATTCAAACAGGAATTGAACCAGAGATTTGGGTTATTCGTGTTGCTACTAGTCCCATACTGTTCTC
AACACTACCAGATGGGAAGATAGTTAAAGGTCTTGCCGGAATCCCAAATGAAGGTCCCGTCTTATTAGTTGGTTA
CCACATGTTGTTGGGATGTGAACTAGGTCCACTTGTTGAAGCATTCTTGAAAGAGAAGAATATTATGATTCGCGG
TGTAGCGCACCCAGAGATGTTTTCAAAGAAGCATGAAGGTCCATCAAATGAATTTGGACCTTTTGATTTGGTGAA
ACTGTTTGGTGGCCTTCCTGTCACACCAAGAAACCTTTTCAAATTGTTTTCAACAAAATCACATATACTCCTTTAT
CCAGGAGGTGCACGTGAGGCTCTTCATCGCAAGGGCGAAAAGTACAAGTTATTGTGGCCCAACCAGCCTGAATT
TGTTAGAATGGCTGCAAGATTTGGTGCCACCATTGTACCGTTTGGGACTGTTGGAGAAGACGACATAGCAGAAG
CGGAATCGACTATGTTGGCAAAGAAGATCACGCTGAGGAGCTCTGACGGCGAGACCTTTGAGGTTGACAAGATC
GTGGCTCTTGAGTCTCAGATGATCAAGCACATGATTGAGGATGACTGTGCGGACAATGGAATACCCTTGCCCAAC
GTCACCAGCTGGATCTTGGTGAAGGTCATCGAGTACTGCAAGAAGCACGTCAAGGCTCCCAAGATCGAGAAAC
GAGGCGACGTCAACAAGGAGCTCAAGTCGTGGGACGCTGAGTTTGTCAAAATTGACTAG

> *MoDGAT2* (Maole_015949), originated from *Malania oleifera*

ATGGCGGAGACCGAGGTCAGAGAGTCGCCGTTGACCGCCGCCCCTGCCCCCGAGGTGACTGTCATCGAGAGCC
CCCGAGTTCCCCTGCTCCATTCCGTGCTCGCCACCGCCCTGTGGATTGGTTGCATCCACCTGTTCTTCGCCGTCAT
GTTCACCGCCACTTTCCTGCTCCCCCTCTCCAAATCCATTGCGGTGTTTGCTCTGCTACTGGTTCTTATTGTGGTTC
CTGTTGAGGCTGATAGTAAATTTGGGATGAAAGTAAGGAGGGGCAATAACCATCATGATAATGAAGGTGATGATG
AAGAGTTCTCCAACATACATGGTTGGGTTGTAAAAAAAAATGGTGGTGGTTTATTGCCACAGTGTCACTATGCCAG
AACTGGAGGTGACAATGCTGCCAAGTAACGGTTCTGTTTTGTCCTTCACAGTCTTTGCGTTGGAGCCACATTCAG
TGTTGCCTGTTGGTGTCATCTCACTTATGCACCTTTCAAATGCTGTGCCCCTCCCAAAAACTAGGGTTCTTGCCAG

TAGCGCTGTGTTTAGAACACCATTCCTGAGGCATATATGGACATGGATGGGCCTTGCAGCTGTAACAAGGAAAAA
TTTTATCTCCCTTTTGGCAGCTGGTTATAGTTGCGCCATAATACCAGGTGGGACGAGGGAGACATTGCTTATGGTG
CAAGATCATGAGGTTTATCTCTTGAAACTCTCTGTGGCTCTATTGCGTATTGCTGCACGCTCTACAGCTTTGGAGT
GGAACTTGTGCCCATTCCAGAAGATTGCTTTCCTTAAGACGAGAAAAGGATTTGTTCGCACGGCTATAGAGACCG
GTGTGCCTCTAATCCCTGTTTTCTCTTTCGGCCAGGTATTTTTTTTTTTTTGGAAAGATCAAGTTTACACCGATGTT
CTTCTGGGGAGTTTTGGGATGCTACATCTCATAACATGGACAGGGCTCCTTTCCACACATCACACACGAATGCCT
TCTTTCTTCATCATGAGGAGAGCTGCCACTGTTCCCCTAACTCCTATGCCTCGTCGGCTTCCATTGCATGTGGTTG
TGGGTAGACCAATTGAGGTTAAGCAAAATTCACAACCCACAGCAGAAGAGGTGAATGAAGTGCACAGTCAGTT
TGTGGGGGCATTGCAAGATCTGTTCGAGAGGCACAAAGCAAGGGTTGGGCACGCTGATCGTGAGCTGAAAATC
ATTTGA

> *MoGPAT* (Maole_006088), originated from *Malania oleifera*

ATGGACTTTGTACCCGACATCTCCGCCATCAAGACCGACAAGATTAAAATTGACAAGGAAACCACCGACATGCT
TACCGCTCGGCATGACGGATCTTCTGAAGTTAGGGAGGACGAGTTGGTGGCTGCGCCAGTGCCAACTTCTCCGT
TCGGTTGTTGGGCCGGCGGACCCGGTAGGAGCATCAACCGTCGCACAAATCAAATACCTCTCGAACCTTGCAGA
GACATCTCAGTTCATAAACTAATCGAACCGATGTGCTCAAAAAGACTAGGGAGTGATTTCTCCGACCAGTTAGCG
TCACCTTGTGTGTCTCATTCGTGGTTGCCGGCGACGACGAAGTGCGATCATGTGCACTGCCTGTGCATGGAACGT
GCCTGCTGCTGGAATTGGTCCTCTGATGATGTGCTTTCAGCTGCTGGCGGATCAAGTCGTGTTGAATCTAATAGTG
TTCATGGCGTATTAAGAAAACTCCAGCAATGGTCTGCAATGAACAGTGCTGCCGATGGGGGGACAAAGGGGCTG
GAATTTGACAGAAAAGACACGAAGAACAAGAATGTGATGAAAGAGAGTACAGTGACCACTCCAAAGACCTTGA
GGGTTGCATTGAAATGCTGCACAAGGGAAGATACTGCACCGGAGGCAAAAGAGAATATGAATTATTTCCGTGGT
ATAGAATGTTGTGAGGTTAAGGAGGCATATTCGAAGGCACTGCATGATTCTGTTACTGAACAATACAATGTGCTC
AAATCTGCCATACATGGCAAACAAGGACTGGAGGCATCCATTCCAGATGTCTCATTATCACAACCATGGCAGTAG

> *MoGPAT*(Maole_006089), originated from *Malania oleifera*

ATGGTGCTGTGGCAGTGCGGTGGGTCTGGACTCAGAGGCAGCGATGAAGGTTTGCGATGGCACGGCGCTGCTAT
GCTTGCAACTCGCACGGTGCCGGCAGCGGCAGGTCTGGACAGTGGACGCCACTCGCACAATGTCCAAGGTAGC
CAACCTCAAATCCAGGAGATCTGTGCGAAAGAGAGCTTGGGGAATATTGACGGTGTGAGGGGTGACTCTATTAA
TGATGCGGAGTGGGCTGGTAAGGCTGTGACCTATGTAGCAGGGGATCGGGTTTTAACGGATATTCTTTGCAAGCC
CTTCAGCATGGGCAGAAATCTTATGTGCGTCTACTCAAAAAAGCACATGCTTGATGATCCTAAGCTTGCTGAGAT
GAAGAAAAAAGCAAATATACGAAGTTTGAAAGAAATGGCCATGCTTTTGAGAGGAGGGTCACAAATAGTATGGA
TTGCACCAAGTGGTGGTAGGGACCGTCCAGATCCCCAGACAGGAGAATGGCATCCGGCACCCTTTGATGCTTCT
TCAGTGGACAATATGAGGCGGCTTGCAGAAAATGCTGGTGTTCCTGCACATATTTATCCATTAGCATTAATTTGCT
ACAACATTATGCCGCCTCCACCCAAGGTTGAGAAAGAGATTGGAGAGAAAAGAGTGGTATCCTTTCATGGGGTT
GGACTATCCGTGATACCAGATATAAGCTATGCTGAAATTGCCGCTGCTTGTGAAAACTCTGAAGAGACTGGCGAT
GCCGACACAAATCAGTCACACTACCAATGTCATCGCAGGATCCCGCAGGCGGAGAAATTTGGCATCTTGGCCGC
ATTGCCCGATGATGAGTCAAGGGCGAAGGCCACATCTCCCAGATCTACGCCATCCAAGCGCCTTCGTTACAAGAT
CGCCGGCTTCTCCGCCCACCTCATGAAGCGAATCCAGAAGGGTCCCTTTCGGGGTATTTCTCTGAAGCTGCAAG
AGGAAGAGCACGAGAGGCGCATGGACTTCGTACCCGACATCTCCGCCATCAAGACCGACAAGATTAAAGTCGA
CAAGGAAACCAGATCTACACCATCCAGGCGCCTTCGCAACAAGATCGCGACTTCTCCACCCACCTCATGA

> *MoGPAT*(Maole_006090), originated from *Malania oleifera*

ATGCAGCTAAGAATACTTTTAGATGTTTTGACTCTTCTGTTTGCAGCATTTCTTTATTTGGAATCTAATTTGGAGGT
ATTGTTTTCTTGTCTTGCTGGGTTTCCTGCAGAGCTGCTTTCTGGAATAAAGAAGGAAACAGAAGCTGGCAGATT
GCCTTCAAATTTTGCTTCAGTTCTGGTTTTCCAAAGTGGAATTCCAGATGCTGATGAAATTATATTGTCAAACACA
ACTGCTTTGTTTGATCGTGTTTTACTGGATGCAGAGGACTCTTTTGTTTTCCCCCCACATCATAAAGCAATGCGAG
AGCCTTTTGATTACTACATGTTTGGTCAAAATTATATCCGTCCTTTGATAGATTTTGGGAATTCATATGTTGGCAATA

TCAACATTTTTCATGAAATGGAAGAGAAGCTGCAGCAGGTCAAGAATCTCAATGGGATTGAGTGGGGTCAGCCA
TGGAGGAGTCTCCACTCTCTGCAAATAGAGAGTAAATCCTTAGATCTGCGACTGGAAAAGGTTGGGAAGGGTTT
CTTTTTGGCTGGATGCTGTTTTCGGTTGCAAAGGGAAATTGTCAAGTTAAATTCTTGGATTTGGGGTTATACTCAT
TTGGAAAGGCTTATTCTATTTTTGTTCTATGGGGTTTTGATGGGGAGTGATGGCAACGGTTTGTTGAGATGTTGCA
GCCGACGGTAA

> *ScINO2*, originated from *Saccharomyces cerevisiae*

ATGCAACAAGCAACTGGGAACGAATTACTGGGTATCCTAGATCTGGATAACGATATAGACTTTGAAACTG
CTTACCAAATGCTCAGCAGTAACTTCGACGACCAAATGTCTGCGCACATACATGAAAACACGTTTAGTGC
AACTTCCCCTCCTCTGTTAACACACGAGCTCGGCATAATTCCTAACGTAGCAACCGTGCAACCCTCTCAC
GTAGAAACTATACCTGCCGATAACCAAACTCATCATGCTCCTTTGCATACTCATGCACACTATCTAAATC
ACAACCCTCATCAACCAAGCATGGGTTTTGATCAAGCGCTTGGTCTCAAGTTGTCTCCTTCCAGTTCGGG
GTTGTTGAGCACGAATGAATCGAATGCCATTGAACAGTTTTTAGACAATCTAATATCACAGGATATGATG
TCTTCCAACGCTTCCATGAACTCCGAATCACATCTACATATAAGATCACCAAAAAAGCAGCATAGGTATA
CCGAATTAAATCAAAGATATCCTGAAACACATCCACACAGTAACACAGGGGAGTTACCCACAAACACAGC
AGATGTGCCAACTGAGTTCACCACGAGGGAAGGACCTCATCAGCCTATCGGCAATGACCACTACAACCCG
CCACCGTTTTCAGTACCTGAGATACGAATCCCAGACTCTGATATTCCAGCCAATATCGAGGACGACCCTG
TGAAGGTACGGAAATGGAAACACGTTCAAATGGAGAAGATACGAAGAATAAACACCAAAGAAGCCTTTGA
AAGGCTCATTAAATCAGTAAGGACCCCACCGAAGGAAAACGGGAAAAGAATTCCCAAGCATATTCTTTTA
ACTTGTGTAATGAACGATATCAAGTCCATTAGAAGCGCAAATGAAGCACTACAGCACATACTGGATGATT
CCTGA

> *YlINO2*, originated from *Yarrowia lipolytic*

ATGTTGAAACCAACCTACATTGAGCCAGCACCATACAAGCTGGAGGAATTGACCATCGACCACAACGTTCCTCA
CTTCAGACAGTCATCCAAGTCCTACAACCCGCATCCCCAGCAGCTAGAGTTCAGAGGAAATTCGTTTGTGTCACT
ACTAAGAGCCCTGGACGGCCGCACCTTGGCGGAGACCCCGAAGTCCTCACCGTGTGTGTCTCCTGAAGTAGAA
AGCTACTTTGCCCCATTAGTCGTTAAGAAACAGCGAAGAGATCGCGTGGTGACAGCTTTCGATACCCTTGAATCC
TCAGAGAGTAACGGCATGTCGCCCCCAATGGTCAACTCGACTAGCCCCATCACTCCAGAATCATCTGGCCTTCAC
AGTACCATTCCGGGAAGTACCCCTTTCGTCGCAGGAAAGAAACCTATTTTAGAACGGGTTTCTGTCAGCAGCGG
GGGACTCCCTGTTGGGAAGCTATTCATGGCAGACATTTGGCAGGATGAGGACCCGGAAACCCCAGACGTGGTGA
ACGTTTTGGTGATGGATTCCGGAAGCCCTGCCGTTGCTACGGCTAGTCCAGGCAAAACTGTGTCTCCATCAGATG
CACTTGACATGATGATCAAATGTGGAGGCAGGGACGCCAGACTCTATCAGGACAGTGCTGCGACGGCTGGGATG
ACCACCACAACCCCCAAGCGCCACCGTGACCAGTGGAAGGCCAAATGGAGTGATACCAAGCGACATAGCAAGG
ACTTCTTCAAGAAAGCCAAAAGAGGTCGAGGAGGAGCTGCATTGAACAGAAGTGACTACTCAGAGTCTGTTTC
TTCCCTTATGGAAGAGACCAACAATAGCTCCGGTGGAGCTAGGGCTCGCTTCAGCGGGGCCAGCGGGGGTAGCG
GGGCCAGCGGGGTTAGTGGTGCCAGCCAGGCTAGCAGTACCAGTGCAGCTACAAGTAGTGTTGCCTGTTCTGAC
AAAGAGGAGGCCAATGGTCCCATTGGTATTAACTTCACTGATTTCAGTGGCGCTTATTCCTACCAGGACATGGTC
ACTGCTAGTGAAGGCACCCCGAGAGCGTGCCACTCGGATCATTTCGGCAGTTTGGAAGAGGACTTGGAGGACG
GCGGAGAAGCCGAGTTGACATCTTCCTACGAGCATTTGTTCGAGAAAGAGTCACTTCGTACCAAGTTTTACAAG
GTGTTCAAGAAGGGGACTTAG

> *YlINO1*, originated from *Yarrowia lipolytic*

ATGCACCTTTCCCACCCACAGGAGAAACAAGTCTTCATCACCACTCCGTCCCCGTCGTCCTCTGTTTCAACCCAG
TCCACCGCCTCGTCGTCACTGTCGCTATTCCACACGGCAGACTCCGATCTCTCCGCCTCCGAAGAAGACGACTTT
CCGTACCACGCAACAACCCACACAAATATAAAGCCTGGTGTGTCTTTCTCCGCCACCTCAGGCACAATGATGAGT
GCGTTATCCCCTGTCTCGGTGCAGTCTCCGTCGACCGTTTTCATGATGAAGGAACCCGCACAGGCACAGGCCCA
GATGTCCCACGACAACAACAACAACAACAACAGTAGCAGCCATGTCACCACCCCTAAACACAACGCCGCCCCT

GTAACCAAATCAGACGACCCCTTCGATGTCGACAACTACCCCACCGACTTTGACTCCGCCTTCCTCAACCTGTCG
CCCACCATGGGGATGGGCCCCGGCGACGGCATGGATGGTATTCCGCTGTTCAACGCAGAGGAGGAGTCCGCTTT
CTCGTCCTTCCTCGACAACGTGGCTCTGGATCCTAACTTCATCTTCGAGCCCAATCTCTCTGATGCCCTGCCCAAG
TGGCCCGAATCGAAACCCTGGGGCCCCAACGACTCGCATAAGCCACGAACCGACAAGTTCACCAAGTTTGGCA
ACTCTCCCGGTCTCGATTTGCTCGCTGCCAATTCCCCCGACGCAGTTAACATCCACGAACGCCTACTGAAACACG
AGTCGAAGGAGTATCTCAGCCCCAACCACAACGACTATGCTCAGAGCGACTCGCGGGCCCAGGCGCTCGCCATG
GCTGAAACACAGGGCTACGGACAGCTAAAACACAAAGAAAGCCGCTCATCGTTATCACAGCCCTCGTCGCTACA
CAACAACAGCGTGACGGGGTCGTCAGTCACAACCACACGTCCCTCGCCACAAACGTCGGGACCCTCCCCCGTG
TCGTCGGGCACGTCAGCGGCGTCGTCCGCACCCATGGGCCTTCATTTCGGCTCAGACCCTGCATTTGGAGGCTCC
AGCTTCCAGCCCAACAGCGGCACCCCAGCCCTCAAAAAGGTGCGGGGCTTCGACGAGCTGCCCCCTGCCGTCC
ACGAGGGACACGGGCACATGCACATGCAGCCACAACGCATGGTGACCGGTCTGGAGGGTGTAGTCAACCCCAA
GCTGGTGGTTGCAGCTGCACAGATGAGCCAGGGCGTGACGGACCAGAACATCTTCATGCTCAAGCGACGAAAG
TCGGACAACATGGCTGCAAGCATGAACTCGAACGTGCCACACGATATCTATGCTCCCGTCCCACATGCTGAGCAG
ATGTATCATATGCAACAACAACAGCAACAGCAGCATCTCCACCAGCAACAGCAGCAACAACATCACCAACAATC
ACAAAATCAGCATATCCAACAACAGCAGCAACAGCAGCAGCATCAGATGCACCACCCTCACCATACACAGCAGC
ACTTCCAGGCACGAATGCACTTTGGCGACGGCATGGATGGCGAGGTGTCCATGAGTACCGTCTCTCAGGCGGGT
TTGCACATGAACTCCAACCCTAGCATGTTGTTCCCCGACAAGGATGCTCTAGCAGACTCGTACCAGCAGCAGCA
GCAGCAAATGCATAGTCAACACAACCCTCCTTCGCATCATCCTCAGCACAACCAGCAGCAGCAGCAGCAACCGC
AGGTCAAGACTGAGCAGAATATGTCTGCATCGCCTACTCCGGGATCTCCCAACCTCACGGAGGACCAGAAACGT
ATGAACCACATCTCTTCCGAAAAGCGACGGCGGGATCTCATCAAGCAGGAGTTTGAGGAAATGTGCGGCCTGGT
GCCTCGTCTGGCTGCAAACAGCGATGAGAAGGGCAAGCGACGCCATGGTCATCGAGGACGTATGCCCAAGGAC
TCGGACAAGGACAAGGATACTGGAACCAAGTCCAAGTCGATTCTACTATCGATTGTGTACGAATACATGTGCGAG
CTGGTGGAGCGAAACAAGGCCATGCGGGGCATGATCACCGAAAAGGGCGGATATCACAGCGATATCGCCAATGC
TCTTCATCCTCCCAAGATTGATGAGTAA

Supplementary Table 4. The amino acid mutations around the binding pocket of AtADS2

**Mutant**	**Mutation site**
AtADS2-M1	G113A, D117F, A210V, E213V, V214Y, C218V
AtADS2-M2	G113A, A210V, E213V, V214Y, C218V
AtADS2-M3	R37Q, R38V, G113A, D117F, A210V, C218V
AtADS2-M4	R37Q, R38V, G113A, D117F, A210V, E213V, V214Y, C218V

Supplementary Table 5. The C16 FAEs and C18 fatty acid desaturases evaluated in substrate specificity in this study

Enzyme	Source	Gene No.	Reference
**C16 FAEs**			
rELO2	*Rattus norvegicus*	NM_134383.2	(Yazawa et al., 2011)
CpLCE	*Cryptosporidium parvum*	AAO34582	(Frltzler et al., 2007)
gELOVL6	*Capra hircus*	NM_001314257.1	(Shi et al., 2017)
**C18 desaturase**			
D9DMB	*Cunninghamella echinulata*	JN873145	(Wan et al., 2013)
CeFAT6	*Caenorhabditis elegans*	NM_001268666.1	(Watts and Browse, 2000)
MaOLE2	*Mortierella alpine*	Y18554.1	(Wongwathanarat et al., 1999)

Supplementary Table 6. Concentration of the media used in central composite design in the first-round experiment

Sources		Lower Limit	Low -1	Center 0	High +1	Higher Limit
Carbon	Glucose (g/L)	46.36	60	80	100	113.64
Nitrogen	Yeast extract (g/L)	1.59	5	10	15	18.41
	Ammonium sulfate (g/L)	-3.41	0	5	10	13.41

Number	Glucose (g/L)	Yeast extract (g/L)	Ammonium sulfate (g/L)	C/N ratio
1	60	5	0	55:1
2	100	5	0	91:1
3	60	15	0	18:1
4	100	15	0	30:1
5	60	5	10	9:1
6	100	5	10	14:1
7	60	15	10	7:1
8	100	15	10	11:1
9	46.36	10	5	9:1
10	113.64	10	5	22:1
11	80	1.59	5	25:1
12	80	18.41	5	11:1
13	80	10	0	36:1
14	80	10	13.41	8:1
15	80	10	5	16:1

Supplementary Table 7. Concentration of the media used in central composite design in the second-round experiment

**Sources**		**Lower Limit**	**Low -1**	**Center 0**	**High +1**	**Higher Limit**
Carbon	Glucose (g/L)	82.96	100	125	150	167.05
Nitrogen	Yeast extract (g/L)	0.64	2	4	6	7.37
	Ammonium sulfate (g/L)	6.64	8	10	12	13.36

**Number**	**Glucose (g/L)**	**Yeast extract (g/L)**	**Ammonium sulfate (g/L)**	**C/N ratio**
1	100	2	8	20
2	150	2	8	29
3	100	6	8	17
4	150	6	8	25
5	100	2	12	13
6	150	2	12	20
7	100	6	12	12
8	150	6	12	18
9	82.96	4	10	12
10	167.05	4	10	25
11	125	0.64	10	21
12	125	7.36	10	17
13	125	4	6.64	26
14	125	4	13.36	14
15	125	4	10	19

Supplementary Figures

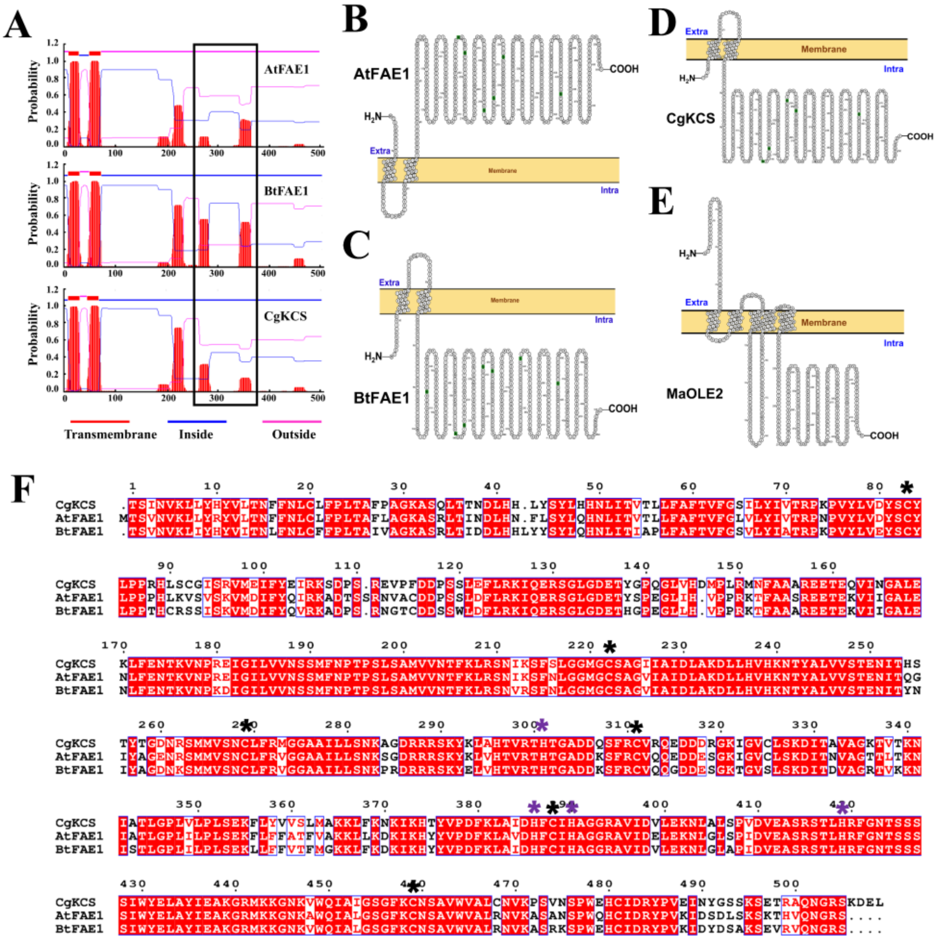

Supplementary Fig. 1. Prediction of transmembrane helices in the fatty acid elongases AtFAE1, BtFAE1, CgKCS and MaOLE2. (A) The pictures showed similar structures among them although they showed distinct capacities on elongating fatty acids in *Y. lipolytica*. The software TMHMM Server v. 2.0 was used. Two-dimensional topology of the entire sequences of AtFAE1 (B), BtFAE1 (C), CgKCS (D) and MaoLE2 (E) constructed by using the online software PROTTER. The catalytic domain of AtFAE1 located in cytosol, while that of the BtFAE1, CgKCS and MaOLE2located in the lumen of ER. (F) Multiple-sequence alignment of fatty acid elongases. CgKCS, AtFAE1 and BtFAE1 have identical conserved cysteines (black asterisk) and histidine residues (purple asterisk).

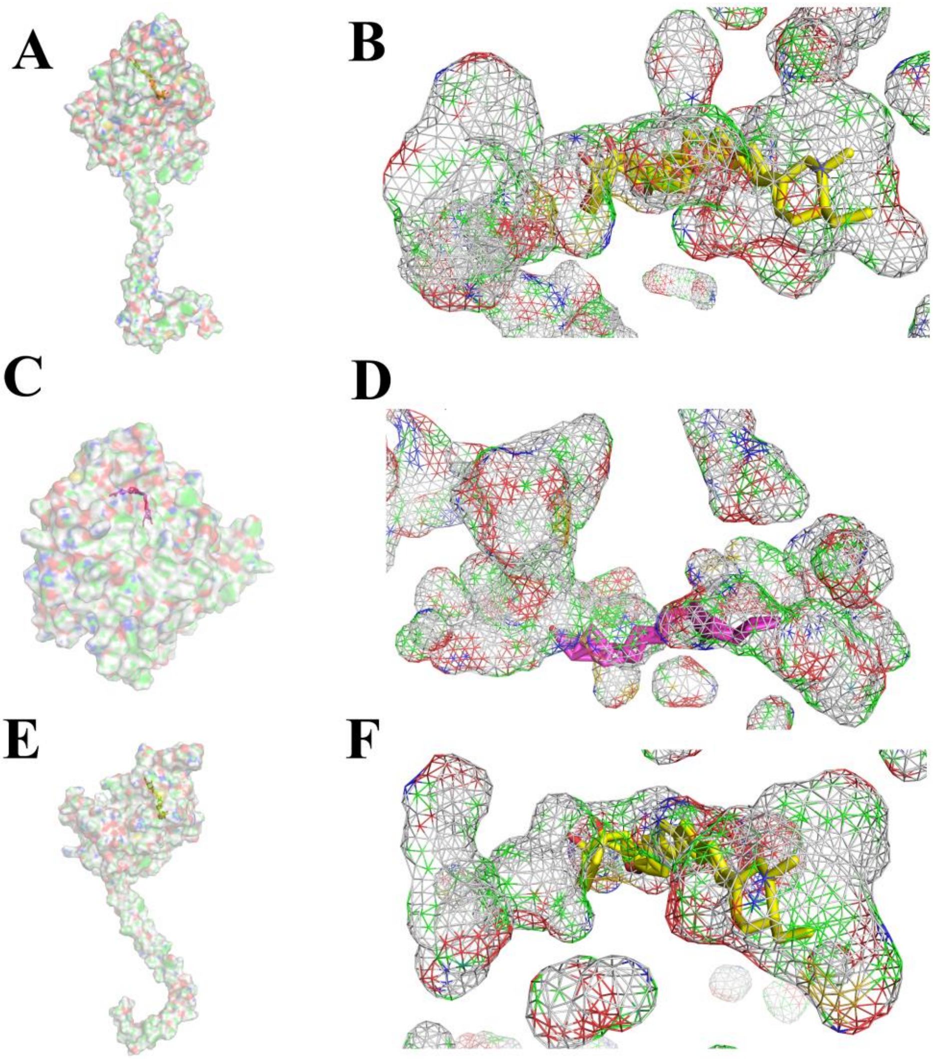

Supplementary Fig. 2. Three-dimensional architectures showing the substrate binding domains in AtFAE1 (A, B), BtFAE1 (C, D) and CgKCS (E, F). The yellow and purple sticks in the catalytic channels denote stearic acid and arachidonic acid, respectively. The enzyme-substrate complexes were modeled according to the crystal structures of *Mycobacterium tuberculosis* PKS11 (PDB ID: 4JAP) for AtFAE1, *Ectocarpus siliculosus* (PDB ID: 4B0N) PKS-I for BtFAE1, and *Mycobacterium tuberculosis* PKS11 (PDB ID: 4JAP) for CgKCS.

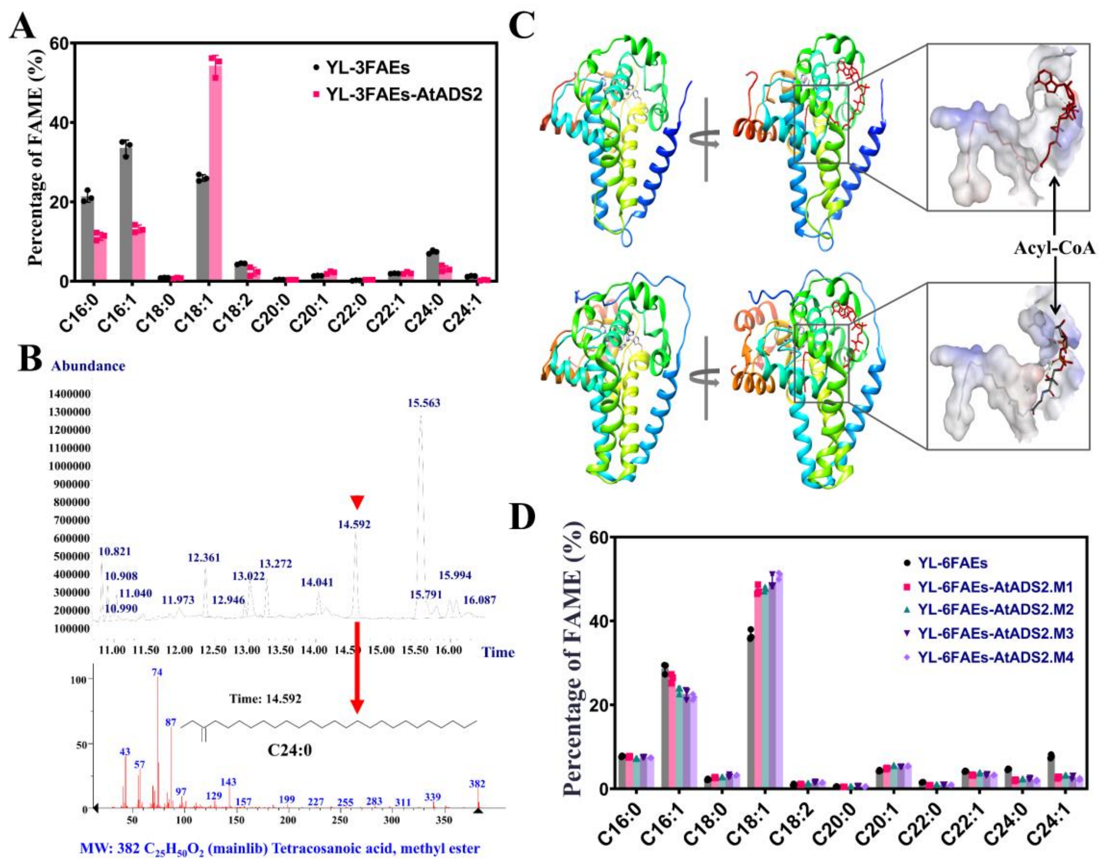

Supplementary Fig. 3. Expression of the acyl-coA desaturase-like protein encoding gene *AtADS2* in *Y. lipolytica*. (A) The production of oleic acid was significantly increased by expressing *AtADS2*. (B) GC-MS detection showed that C24:0-CoA was not desaturated to form C24:1-CoA. (C) Three-dimensional architecture of the C18-acyl-CoA binding domain. The up and down pictures denote AtADS2 and mouse SCD, respectively. The model of the AtADS2/C18-acyl-CoA complex was created by superimposing AtADS2 to the mouse SCD (PDB ID: 4ymk). (D) Expression of *AtADS2* mutants in the strain YLVL6 significantly increased the production of oleic acid but decreased the content of nervonic acid in total fatty acids (TFA).

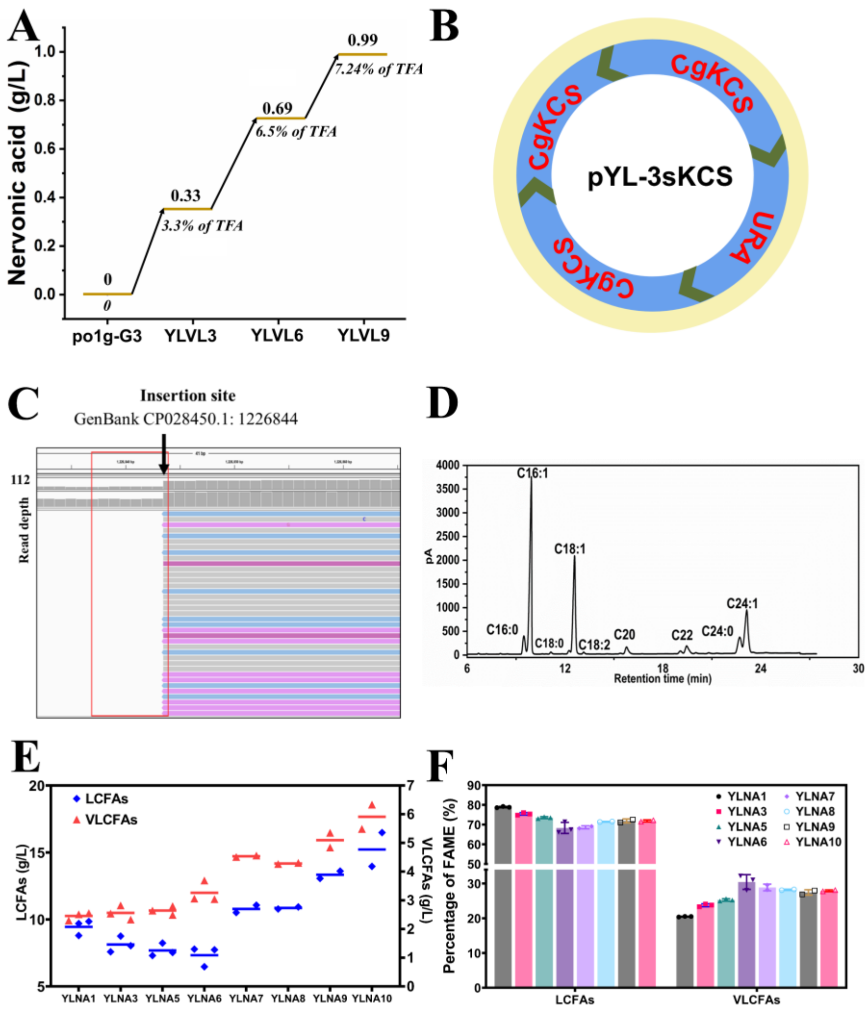

Supplementary Fig. 4. Increasing nervonic acid production by random genetic insertion and homologous recombination. (A) Nervonic acid production increased by iterative expression of *CgKCS*. (B) The arrangement of three copies of CgKCS in the plasmid YL-3sKCS. (C) Identifying the insertion sites of the *CgKCS* expression cassettes by genome resequencing. The picture was generated by the Integrative Genomics Viewer (IGV) software. The insertion site CP028450.1: 1226844 located in the elongation factor 1-alpha (TEF1) gene in *Y. lipolytica*. The results of lipid and VLCFAs biosynthesis in the strains YLNA1, 3, and 5 to 10, including the fatty acid composition in YLNA9 (D), the titer of LCFAs and VLCFAs (E), and the fraction of LCFAs and VLCFAs (F).

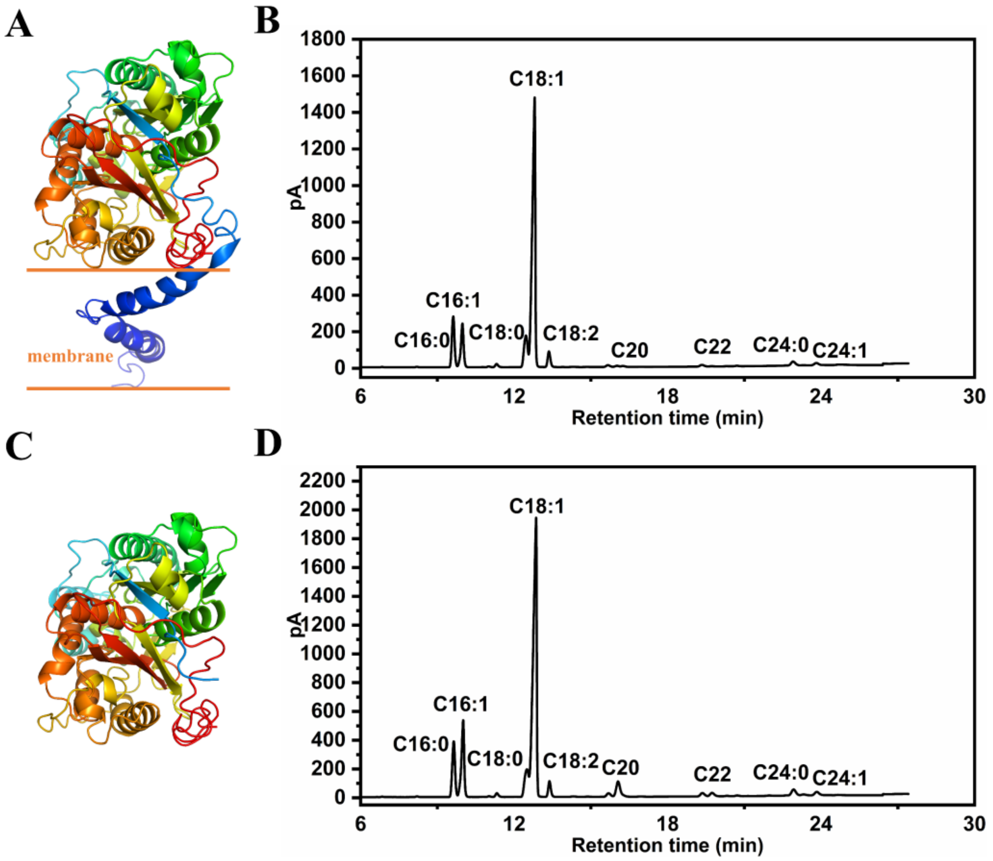

Supplementary Fig. 5. The influence of the transmembrane region on the catalytic ability of CgKCS. The structural models of CgKCS (A) and the truncated CgKCS (C). The fraction of fatty acids in the control strain (B) and the truncated CgKCS expressing strain (D).

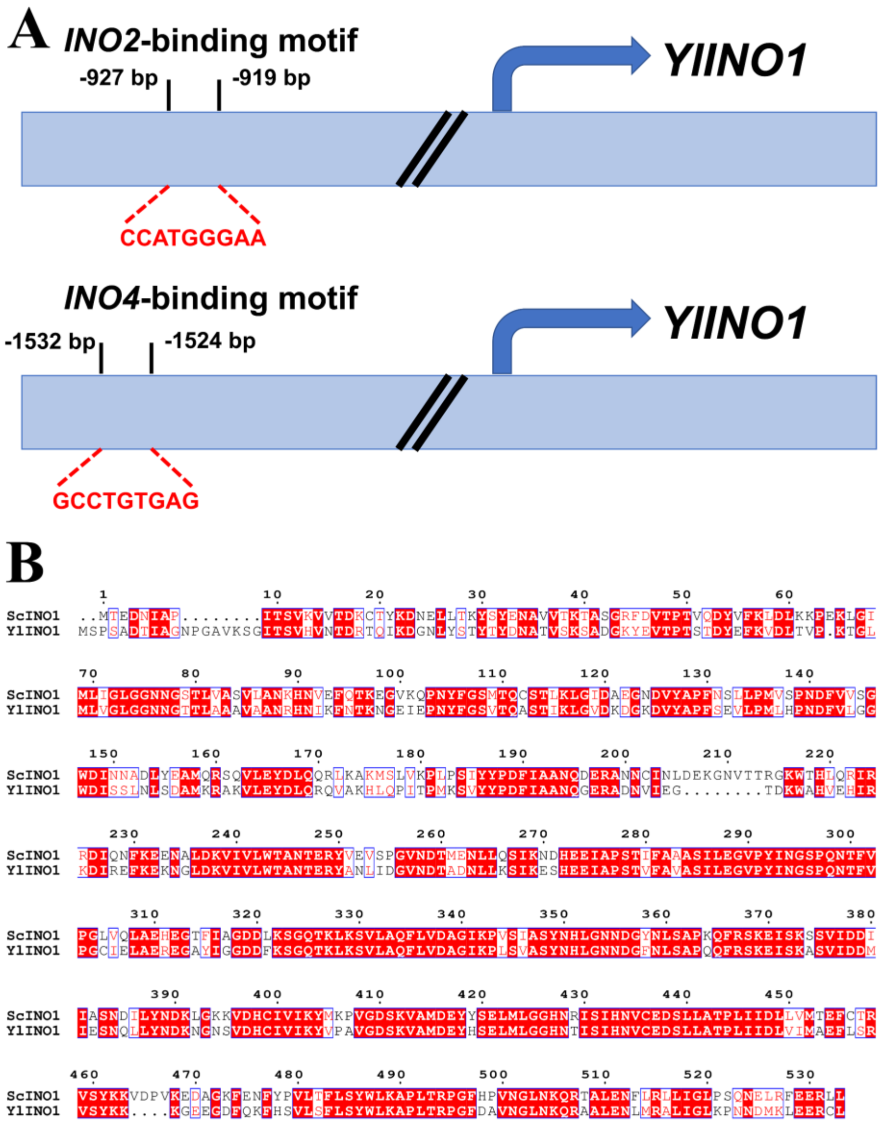

Supplementary Fig. 6. Predicting the interaction of *S. cerevisiae* INO2/INO4 with the promoter of the *Y. lipolytica INO1*. (A) Schematic diagram showing the ScINO2 and ScINO4 binding motifs in the YlINO1 promoter region. (B) Multiple-sequence alignment of INO1 proteins. Sc, *Saccharomyces cerevisiae*; Yl, *Yarrowia lipolytica*. Conserved amino acids are denoted in red boxes.

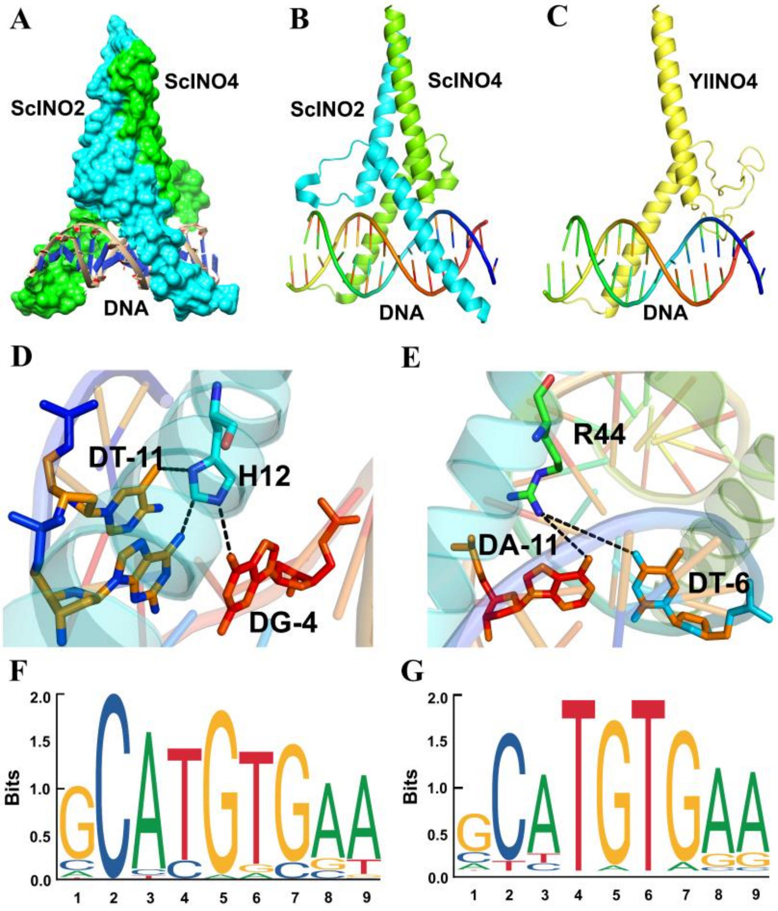

Supplementary Fig. 7. Three-dimensional architecture and sequence frequency analysis of ScINO2/ScINO4. (A) and (B) Overall three-dimensional structural model of the surface of INO2/INO4 complex. (C) Three-dimensional architecture of the complex of YlINO4 combining with DNA. (D) and (E) The interaction between ScINO2 and the promoter. Black dash lines denote the interaction between critical amino acid residues and nucleic acids. The sequence frequency of ScINO2 (F) and ScINO4 (G).

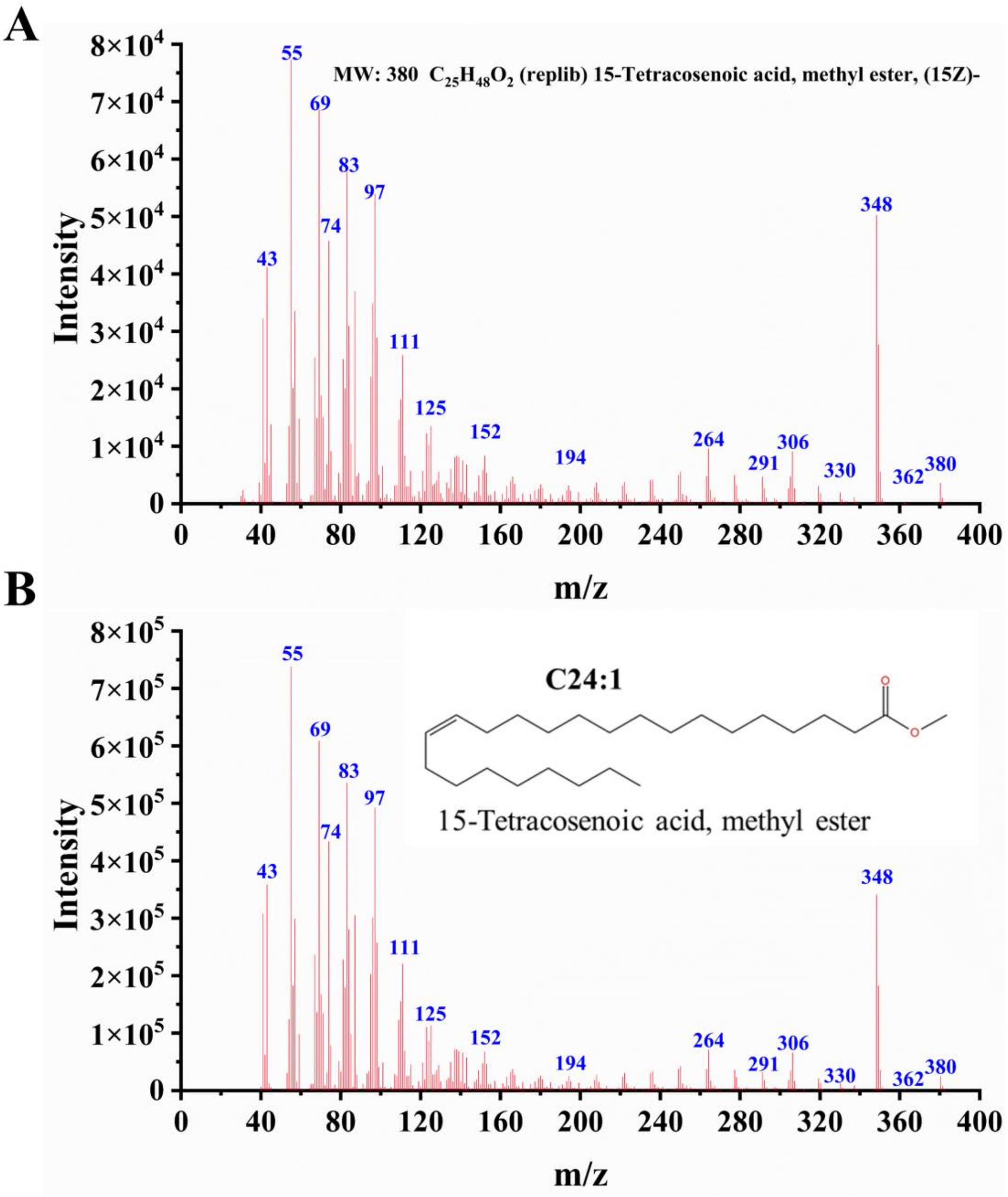

Supplementary Fig. 8. Identification of nervonic acid in the lipids produced by the engineered *Y. lipolytica* strains by GC-MS. The mass spectra of nervonic acid in standard (A) and the samples (B).

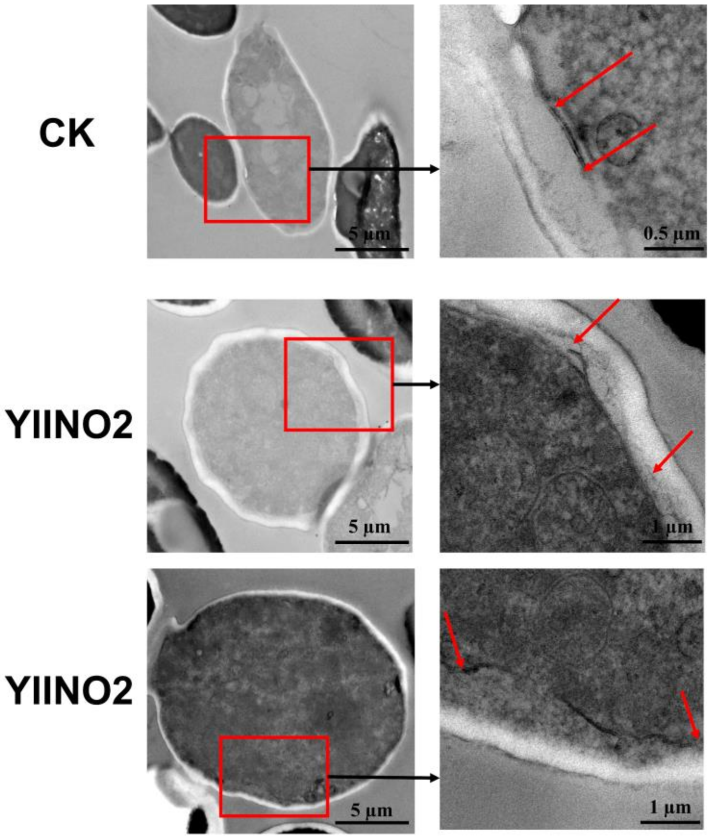

Supplementary Fig. 9. Overexpression of YlINO2 enlarged the size of ER. Transmission electron micrographs of the CK (YLNA8) and YlINO2 (YLNA9) strains. The red arrows indicate the two ends of ER.

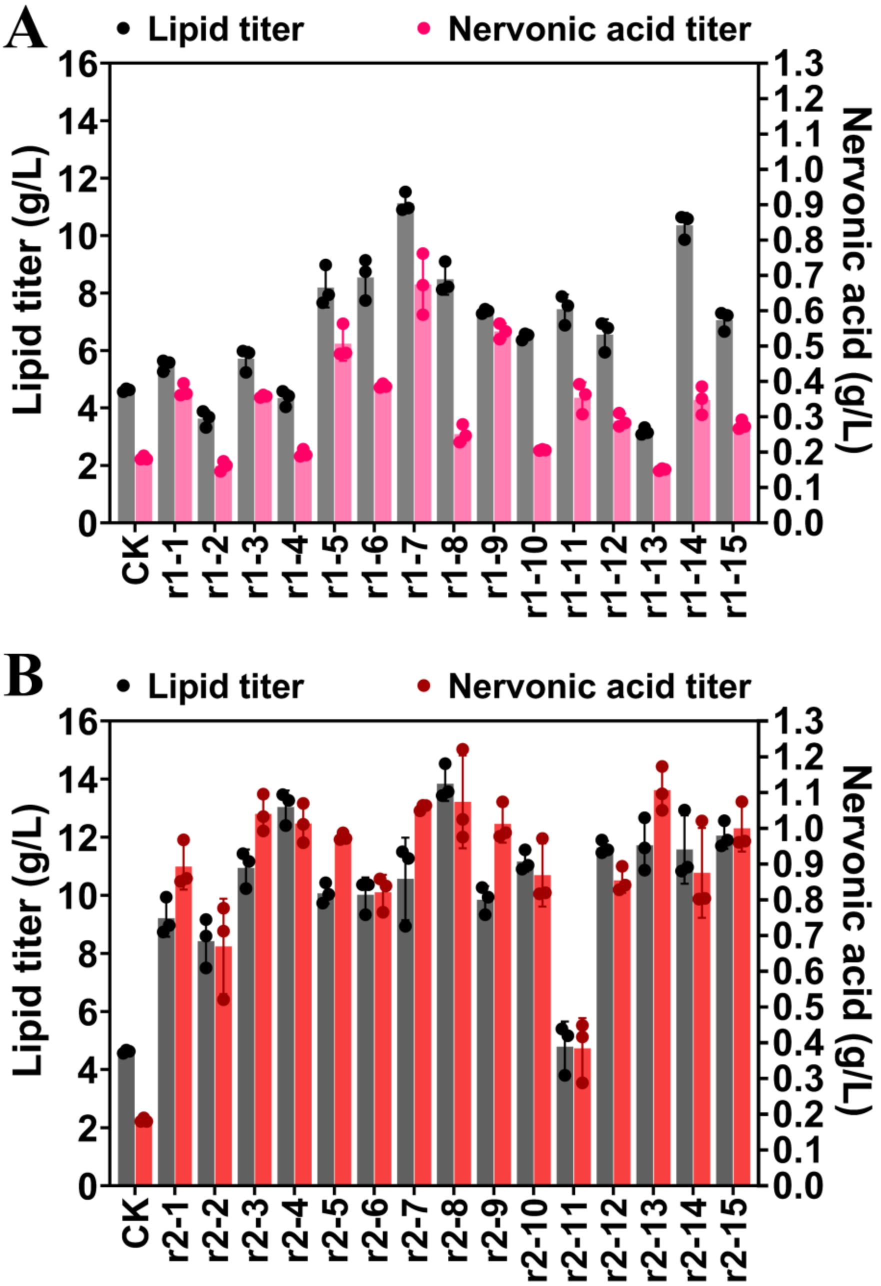

Supplementary Fig. 10. Lipid and nervonic acid production by the strain YLVL3 in different media designed by the central composite design (CCD) and response surface methodology. (A) The first round of medium optimization. The medium r1-7 was used in next optimization. (B) The second round of medium optimization. The medium r2-8 was used in the following fermentation.

A

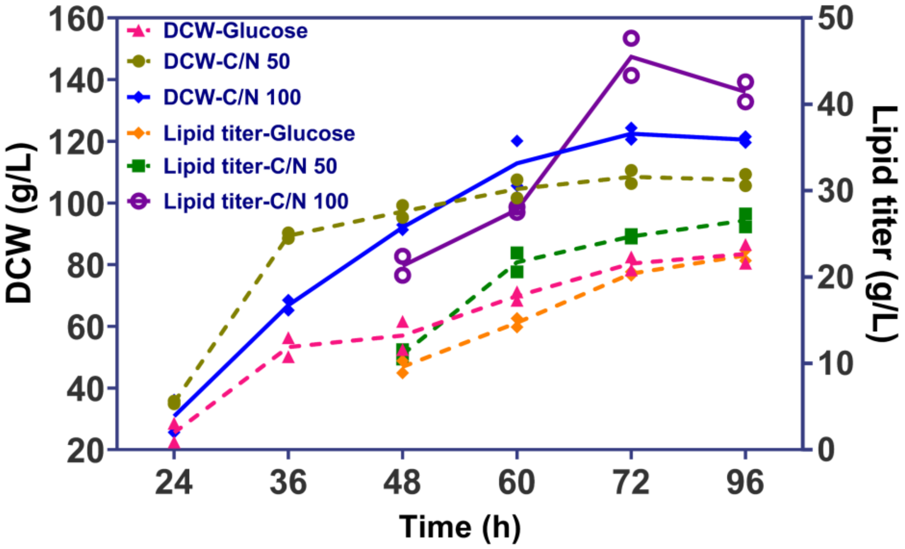

B

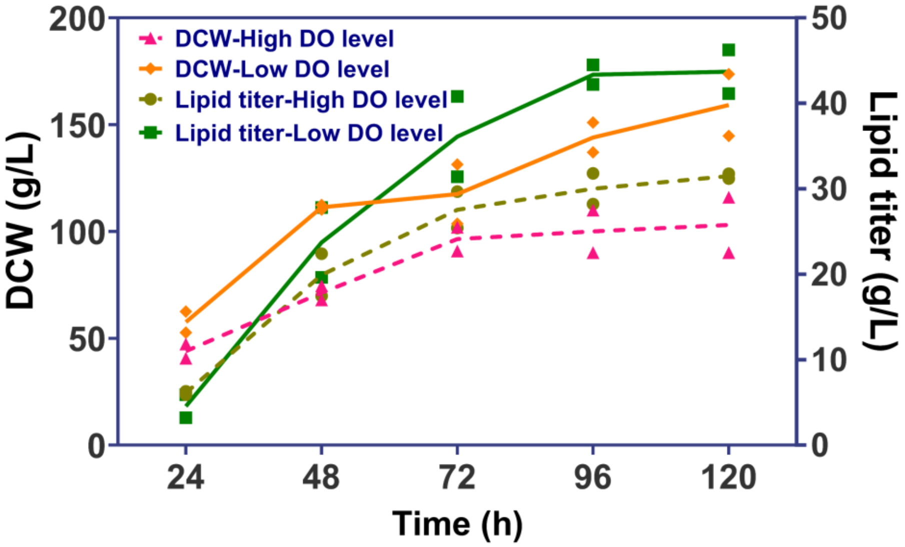

Supplementary Fig. 11. Optimization of feeding medium composition (A) and dissolved oxygen level (B) in a 3-L reactor. Feeding glucose and ammonium sulfate with a C/N molar ratio of 100:1 during fermentation resulted in an 84.6% increase in lipid titer compared to feeding sole glucose (A). The lipid titer was further improved to 43.7 g/L by controlling the dissolved oxygen level at 20% in 24 h and below 5% after 24 h (B). Data are mean ± s.d. from two replicates.

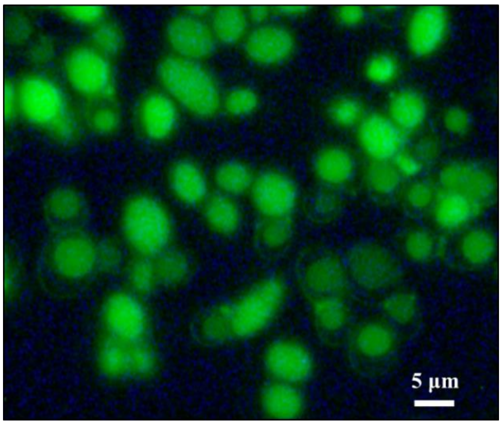

Supplementary Fig. 12. Observation of the lipid accumulation in the strain YLNA9 by Nile red dyeing. The cells cultivated for 216 h in the 50-L reactor was used. The green color represents the lipid droplets in cells.

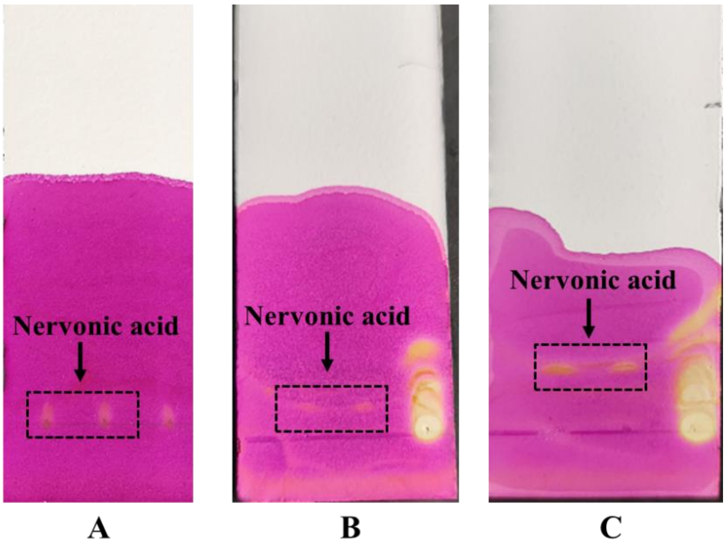

Supplementary Fig. 13. Evaluation of the elution buffers used in silica gel column chromatography for nervonic acid separation by thin layer chromatography (TLC). The developing buffers included *n*-hexane supplemented with 0.5% ammonia (A), 0.5% acetic acid (B), and 1% acetic acid (C). The outcome showed that the buffer consisting of *n*-hexane and 1% acetic acid exhibited best separation efficiency and was used in silica gel column chromatography.

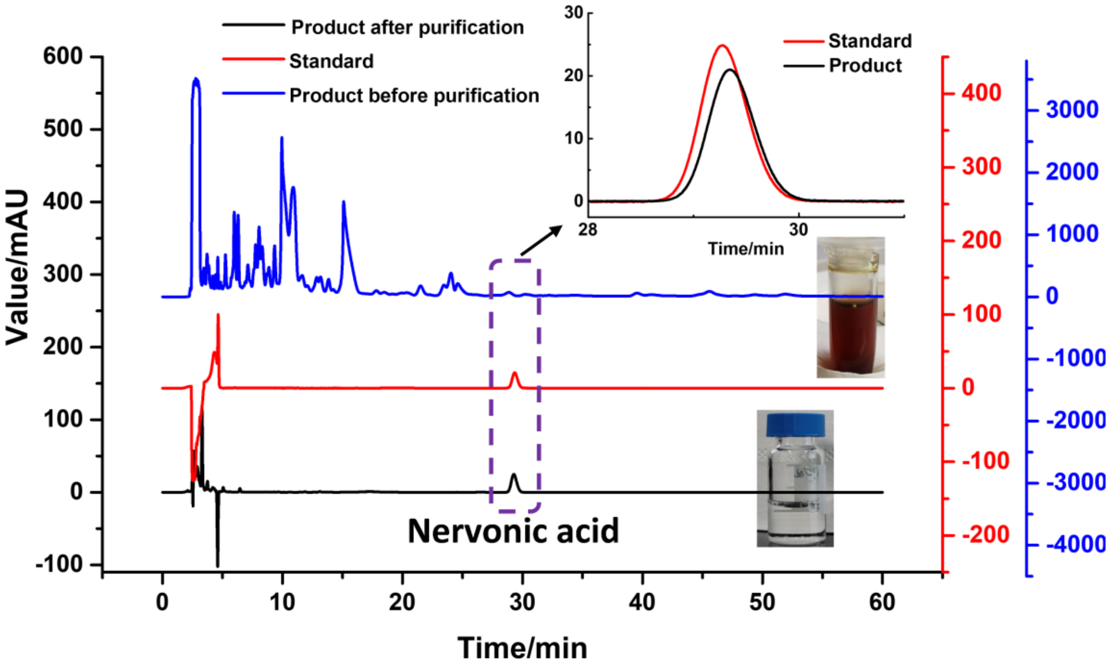

Supplementary Fig. 14. Comparison of nervonic acid products before and after purification determined by HPLC. The brown liquid showed the initial lipids. The transparent liquid presented the purified nervonic acid solution.

Supplementary References

Frltzler, J.M., Millership, J.J., Zhu, G., 2007. *Cryptosporidium parvum* long-chain fatty acid elongase. Eukary. Cell. 6, 2018-2028.

Li, J.X., Xu, J., Ruan, J.C., Meng, H.M., Su, H., Han, X.F., et al., 2020. Disrupting a phospholipase A2 gene increasing lipid accumulation in the oleaginous yeast *Yarrowia lipolytica*. J. Appl. Microbiol. 130, 100-108.

Shi, H.B., Wu, M., Zhu, J.J., Zhang, C.H., Yao, D.W., Luo, J., Loor, J.J., 2017. Fatty acid elongase 6 plays a role in the synthesis of long-chain fatty acids in goat mammary epithelial cells. J. Dairy. Sci. 100, 4987-4995.

Wan, X., Liang, Z., Gong, Y., Zhang, Y., Jiang, M., 2013. Characterization of three Delta9-fatty acid desaturases with distinct substrate specificity from an oleaginous fungus *Cunninghamella echinulata*. Mol. Biol. Rep. 40, 4483-4489.

Watts, J.L., Browse, J., 2000. A palmitoyl-CoA-specific Δ9 fatty acid desaturase from *Caenorhabditis elegans*. Biochem. Biophys. Res. Commun. 272, 263-269.

Wongwathanarat, P., Michaelson, L.V., Carter, A.T., Lazarus, C.M., Griffiths, G., Stobart, A.K., et al., 1999. Two fatty acid Δ9-desaturase genes, *ole1* and *ole2*, from *Mortierella alpina* complement the yeast *ole1* mutation. Microbiol-SGM. 145, 2939-2946.

Yazawa, H., Kamisaka, Y., Kimura, K., Yamaoka, M., Uemura, H., 2011. Efficient accumulation of oleic acid in *Saccharomyces cerevisiae* caused by expression of rat elongase 2 gene (*rELO2*) and its contribution to tolerance to alcohols. Appl. Microbiol. Biot. 91, 1593-1600.
