# Supplementary material for: High-level production of nervonic acid in the oleaginous yeast Yarrowia lipolytica by systematic metabolic engineering
Source: Commun Biol. 2023 Nov 7;6:1125. doi: 10.1038/s42003-023-05502-w (PMC10630375; doi:10.1038/s42003-023-05502-w)
Supplement: Supplementary file 3 — Description of Additional Supplementary Files [file 42003_2023_5502_MOESM3_ESM.pdf]

Description of Additional Supplementary Files

File name: Supplementary Data 1
Description: Primers used for construction of plasmids.

File name: Supplementary Data 2
Description: Source data.
