# Supplementary material for: High-level production of nervonic acid in the oleaginous yeast Yarrowia lipolytica by systematic metabolic engineering
Source: Commun Biol. 2023 Nov 7;6:1125. doi: 10.1038/s42003-023-05502-w (PMC10630375; doi:10.1038/s42003-023-05502-w)
Supplement: Supplementary file 6 — Reporting Summary [file 42003_2023_5502_MOESM6_ESM.pdf]

nature portfolio

Corresponding author(s): Shi'an Wang, Fuli Li

Last updated by author(s): Oct 16, 2023

Reporting Summary

Nature Portfolio wishes to improve the reproducibility of the work that we publish. This form provides structure for consistency and transparency in reporting. For further information on Nature Portfolio policies, see our Editorial Policies and the Editorial Policy Checklist.

Statistics

For all statistical analyses, confirm that the following items are present in the figure legend, table legend, main text, or Methods section.

n/a	Confirmed	
☐	☒	The exact sample size (*n*) for each experimental group/condition, given as a discrete number and unit of measurement
☒	☐	A statement on whether measurements were taken from distinct samples or whether the same sample was measured repeatedly
☒	☐	The statistical test(s) used AND whether they are one- or two-sided<br>*Only common tests should be described solely by name; describe more complex techniques in the Methods section.*
☒	☐	A description of all covariates tested
☒	☐	A description of any assumptions or corrections, such as tests of normality and adjustment for multiple comparisons
☒	☐	A full description of the statistical parameters including central tendency (e.g. means) or other basic estimates (e.g. regression coefficient) AND variation (e.g. standard deviation) or associated estimates of uncertainty (e.g. confidence intervals)
☒	☐	For null hypothesis testing, the test statistic (e.g. *F*, *t*, *r*) with confidence intervals, effect sizes, degrees of freedom and *P* value noted<br>*Give P values as exact values whenever suitable.*
☒	☐	For Bayesian analysis, information on the choice of priors and Markov chain Monte Carlo settings
☒	☐	For hierarchical and complex designs, identification of the appropriate level for tests and full reporting of outcomes
☒	☐	Estimates of effect sizes (e.g. Cohen's *d*, Pearson's *r*), indicating how they were calculated

Our web collection on statistics for biologists contains articles on many of the points above.

Software and code

Policy information about availability of computer code

Data collection	For membrane protein topology visualization, the PROTTER online servers was used (Omasits et al., 2014). Structure homology modeling of MaOLE2 was carried out using the SWISS-MODEL online servers (Schwede et al., 2003). The three-dimensional structure of a mouse stearoyl-CoA desaturase (PBD ID: 4YMK) in PDB was used as the template for homology modeling of MaoLE2. Three-dimensional structures modeling of CgKCS was carried out using I-TASSER unified platform (Yang et al., 2015). The predicted protein structures were visualized using the program UCSF Chimera (Pettersen et al., 2004) and PyMOL Molecular Graphics System (Version 1.7.0.0).

Data analysis

1. To evaluate the substrate preference of AtADS2, the 3D structural model of AtADS2 was constructed by SWISS-MODEL server using mouse SCD (PDB ID: 4ymk) as the template (Schwede et al., 2003). Pictures of AtADS2 structures were generated with the program UCSF Chimera (Pettersen et al., 2004). The docking of acyl-CoA into the binding pocket of AtADS2 was performed with the AutoDock Vina software (Trott et al., 2010). The 3D structures of acyl-CoA and the minimized energy structures were constructed by BIOVIA Discovery Studio 4.5. Next, AutoDock Tools 1.5.4 was used to assign hydrogens, Gasteiger charges and rotatable bonds to acyl-CoA.
2. The genome sequences of representative edited clones were sequenced by Illumina technique. Standard genome sequencing and standard bioinformatic analyses were provided by Oebiotech (Shanghai, China). The filtered reads were mapped to the reference genome Y. lipolytica CLIB89 (W29) (GenBank: GCA_001761485.1) using the BWA 0.7.16a software. The aligned sequence reads were visualized by Integrative Genomics Viewer (IGV).
3. Homology modeling of the retrieved YlINO2 and YlINO4 from Y. lipolytica was performed using SWISS-MODEL server (Schwede et al., 2003). The bHLH ScINO2-ScINO4 transcription activation complex bound the promoter region (PDB ID: 7XQ5) and the structures of TFIIIB-TBP/Brf2/DNA and SANT domain of Bdp1 (PDB ID: 5N9G) in Protein Databank (PDB) were used as templates for homology modeling of YlINO4 and YlINO2, respectively. The predicted protein structures were visualized using PyMOL Molecular Graphics System (Version 1.7.0.0). Figure 2F, 6A, 7A, 7B and 8A were created by Figdraw (www.figdraw.com). Structure-based multiple sequence alignment of the bHLH transcription factors was carried out on the T-COOFFEE online service (Notredame et al., 2000).

For manuscripts utilizing custom algorithms or software that are central to the research but not yet described in published literature, software must be made available to editors and reviewers. We strongly encourage code deposition in a community repository (e.g. GitHub). See the Nature Portfolio guidelines for submitting code & software for further information.

Data

Policy information about availability of data

All manuscripts must include a data availability statement. This statement should provide the following information, where applicable:

- Accession codes, unique identifiers, or web links for publicly available datasets
- A description of any restrictions on data availability
- For clinical datasets or third party data, please ensure that the statement adheres to our policy

The authors declare that the data supporting the findings of this study are available within the Article and its Supplementary Information files.

Research involving human participants, their data, or biological material

Policy information about studies with human participants or human data. See also policy information about sex, gender (identity/presentation), and sexual orientation and race, ethnicity and racism.

Reporting on sex and gender

Use the terms sex (biological attribute) and gender (shaped by social and cultural circumstances) carefully in order to avoid confusing both terms. Indicate if findings apply to only one sex or gender; describe whether sex and gender were considered in study design; whether sex and/or gender was determined based on self-reporting or assigned and methods used.
Provide in the source data disaggregated sex and gender data, where this information has been collected, and if consent has been obtained for sharing of individual-level data; provide overall numbers in this Reporting Summary. Please state if this information has not been collected.
Report sex- and gender-based analyses where performed, justify reasons for lack of sex- and gender-based analysis.

Reporting on race, ethnicity, or other socially relevant groupings

Please specify the socially constructed or socially relevant categorization variable(s) used in your manuscript and explain why they were used. Please note that such variables should not be used as proxies for other socially constructed/relevant variables (for example, race or ethnicity should not be used as a proxy for socioeconomic status).
Provide clear definitions of the relevant terms used, how they were provided (by the participants/respondents, the researchers, or third parties), and the method(s) used to classify people into the different categories (e.g. self-report, census or administrative data, social media data, etc.)
Please provide details about how you controlled for confounding variables in your analyses.

Population characteristics

Describe the covariate-relevant population characteristics of the human research participants (e.g. age, genotypic information, past and current diagnosis and treatment categories). If you filled out the behavioural & social sciences study design questions and have nothing to add here, write "See above."

Recruitment

Describe how participants were recruited. Outline any potential self-selection bias or other biases that may be present and how these are likely to impact results.

Ethics oversight

Identify the organization(s) that approved the study protocol.

Note that full information on the approval of the study protocol must also be provided in the manuscript.

Field-specific reporting

Please select the one below that is the best fit for your research. If you are not sure, read the appropriate sections before making your selection.

☒ Life sciences ☐ Behavioural & social sciences ☐ Ecological, evolutionary & environmental sciences

For a reference copy of the document with all sections, see nature.com/documents/nr-reporting-summary-flat.pdf

Life sciences study design

All studies must disclose on these points even when the disclosure is negative.

Sample size	The yeasts were cultured for 144 h at 250 rpm and 28 ºC in flasks. Data are mean ± s.d. from three or two replicates.
Data exclusions	No date were excluded from the analyses.
Replication	All attempts at replication were successful.
Randomization	After expression of genes by homologous recombination at the specific gene loci, clones were randomly allocated and positive clones were verified by colony PCR and DNA sequencing.
Blinding	Investigators were blinded to group allocation and analysis.

Reporting for specific materials, systems and methods

We require information from authors about some types of materials, experimental systems and methods used in many studies. Here, indicate whether each material, system or method listed is relevant to your study. If you are not sure if a list item applies to your research, read the appropriate section before selecting a response.

Materials & experimental systems

n/a	Involved in the study
☒	☐ Antibodies
☒	☐ Eukaryotic cell lines
☒	☐ Palaeontology and archaeology
☒	☐ Animals and other organisms
☒	☐ Clinical data
☒	☐ Dual use research of concern
☒	☐ Plants

Methods

n/a	Involved in the study
☒	☐ ChIP-seq
☒	☐ Flow cytometry
☒	☐ MRI-based neuroimaging

Plants

Seed stocks	*Report on the source of all seed stocks or other plant material used. If applicable, state the seed stock centre and catalogue number. If plant specimens were collected from the field, describe the collection location, date and sampling procedures.*
Novel plant genotypes	*Describe the methods by which all novel plant genotypes were produced. This includes those generated by transgenic approaches, gene editing, chemical/radiation-based mutagenesis and hybridization. For transgenic lines, describe the transformation method, the number of independent lines analyzed and the generation upon which experiments were performed. For gene-edited lines, describe the editor used, the endogenous sequence targeted for editing, the targeting guide RNA sequence (if applicable) and how the editor was applied.*
Authentication	*Describe any authentication procedures for each seed stock used or novel genotype generated. Describe any experiments used to assess the effect of a mutation and, where applicable, how potential secondary effects (e.g. second site T-DNA insertions, mosiacism, off-target gene editing) were examined.*
